# Thermal Half-Lives of Azobenzene Derivatives: Virtual Screening Based on Intersystem Crossing Using a Machine Learning Potential

**DOI:** 10.1021/acscentsci.2c00897

**Published:** 2023-01-25

**Authors:** Simon Axelrod, Eugene Shakhnovich, Rafael Gómez-Bombarelli

**Affiliations:** † Department of Chemistry and Chemical Biology, Harvard University, Cambridge, Massachusetts02138, United States; ‡ Department of Materials Science and Engineering, Massachusetts Institute of Technology, Cambridge, Massachusetts02139, United States

## Abstract

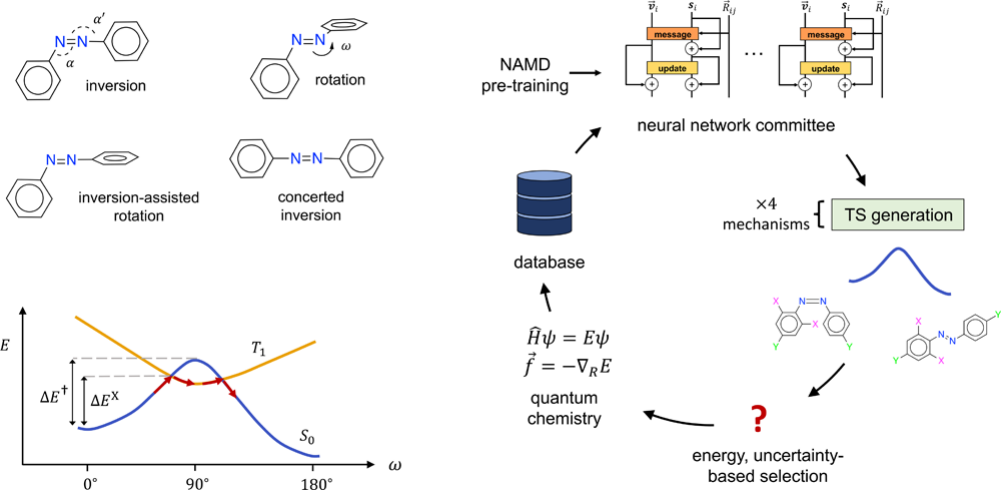

Molecular photoswitches are the foundation of light-activated drugs. A key photoswitch is azobenzene, which exhibits *trans*–*cis* isomerism in response to light. The thermal half-life of the *cis* isomer is of crucial importance, since it controls the duration of the light-induced biological effect. Here we introduce a computational tool for predicting the thermal half-lives of azobenzene derivatives. Our automated approach uses a fast and accurate machine learning potential trained on quantum chemistry data. Building on well-established earlier evidence, we argue that thermal isomerization proceeds through rotation mediated by intersystem crossing, and incorporate this mechanism into our automated workflow. We use our approach to predict the thermal half-lives of 19,000 azobenzene derivatives. We explore trends and trade-offs between barriers and absorption wavelengths, and open-source our data and software to accelerate research in photopharmacology.

## Abstract 2

We develop an automated tool for predicting thermal half-lives of azobenzene derivatives using a machine learning potential. Our method also accounts for isomerization through intersystem crossing.

## Introduction

Photoswitches are compounds whose properties can be modified by light. They have applications in many developing technologies, such as organic electronics,^1^ energy storage,^2^ and targeted medicine.^3^ The latter includes photopharmacology, the field of light-activated drugs. Such drugs are built around photoswitchable scaffolds, which allows their medicinal activity to be controlled with light. The most common scaffold is azobenzene, which undergoes *cis* ↔ *trans* isomerization in response to light. An inactive drug built around azobenzene can be activated with light at certain times or in certain regions of the body. This can minimize off-target activity, thereby minimizing side effects.^3^

The development of photoactive drugs is a complex, multiobjective optimization problem. A formidable number of properties must be optimized for all drugs. Photoactive compounds must also absorb light at the right wavelength, typically in the near-infrared region; isomerize with high efficiency in the excited state; display differential bioactivity between the two isomers; and thermally revert to the stable isomer in a specific time frame.^4^ For many applications this time frame should be as long as possible, and so the isomerization barrier should be as high as possible.^4^ Yet substituents that shift absorption from the UV to the visible or near-IR regions often lower the thermal barrier.^3^ This highlights the challenge of the optimization problem.

The design of photoactive drugs can be accelerated with computational modeling. Property predictors can be applied to large virtual libraries, and the results can be used to narrow the search space of promising compounds.^5^ Quantum chemistry can predict many properties with good accuracy, but the calculations are quite slow. In our previous work, we showed how a machine learning (ML) potential trained on quantum chemistry data can be used to rapidly predict the quantum yield of azobenzene derivatives.^6^ In this work we develop an ML-based computational workflow to predict the thermal half-lives of azobenzene derivatives.

Our contributions are as follows. First, we improve the theory of thermal azobenzene isomerization. In particular, we provide evidence that thermal isomerization proceeds through intersystem crossing, not through a typical singlet transition state (TS). This builds on the theory that was proposed nearly 20 years ago,^7^ but which has largely been overlooked (with some exceptions^8,9^). We also demonstrate the critical importance of multireference effects in barrier calculations.

Second, we provide a fast and user-friendly computational tool for predicting the barriers and absorption wavelengths of azobenzene derivatives. Our program can be run with a single command. The only user input required is the SMILES strings of the relevant compounds. These can be generated programatically, or with visual interface programs such as ChemDraw. Further, the program is quite fast, replicating the results of spin-flip, time-dependent density functional theory (SF-TDDFT)^10^ in milliseconds through use of a transferable ML potential. This program adds to the growing collection of computational models for predicting photoswitch properties.^6,11−13^ Our software and pretrained models are freely available at https://github.com/learningmatter-mit/azo_barriers.

Third, we use our tool to perform virtual screening of nearly 19,000 hypothetical azobenzene derivatives. We identify species with high isomerization barriers and red-shifted absorption spectra. The data is freely available at DOI 10.18126/unc8-336t through the Materials Data Facility.^14,15^ Researchers can use the species with favorable properties as scaffolds for new photoactive drugs. Further, we explain these results in terms of substitution patterns and substituent properties. These insights will accelerate the design of metastable, red-shifted azobenzene derivatives in the future.

## Theory and Methods

### Isomerization Mechanisms

**Figure 1:**
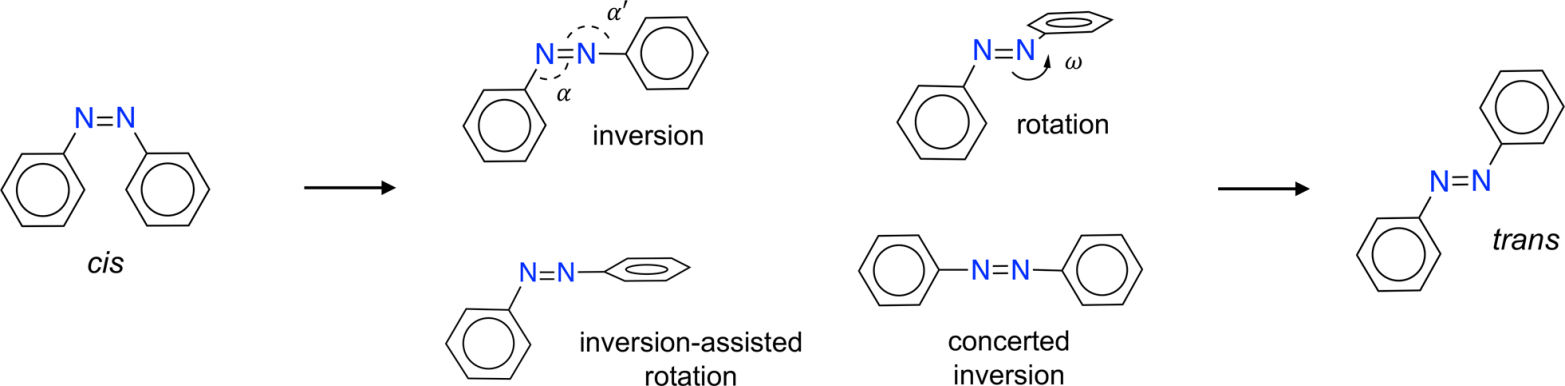
Possible mechanisms of thermal isomerization. The inversion TS has α or α′ ≈ 180°, while the rotational TS has ω ≈ 90°. Inversion-assisted rotation combines inversion and rotation. Concerted inversion combines α inversion and α′ inversion.

Four mechanisms have been proposed for azobenzene isomerization:^16^ rotation, inversion, inversion-assisted rotation, and concerted inversion (Figure 1). Rotation is characterized by ω ≈ 90° and α ≈ α′ ≈ 120°. Both inversion mechanisms have α ≈ 180° and α′ ≈ 120°; pure inversion has ω ≈ 180°, while inversion-assisted rotation has ω ≈ 90°. (Some works refer to inversion-assisted rotation simply as rotation,^11^ but we avoid that terminology here.) Concerted inversion has α ≈ α′ ≈ 180°, but can be excluded from possible thermal mechanisms; see the Supporting Information (SI) Section S11.1. Here we group together inversion and inversion-assisted rotation, and refer to both as inversion (Section S11.2). For asymmetrically substituted azobenzenes, α = 180° is distinct from α′ = 180°, and ω = 90° is distinct from ω = −90°. This gives two inversion TSs and two rotational TSs.

### Standard Models

Several works have predicted the thermal lifetimes of azobenzene derivatives with computational methods.^11,12,17−20^ However, there are two main issues with previous calculations. First, all levels of theory overestimate the experimental enthalpy and entropy of activation. The experimental activation entropy is −50.2 J mol^–1^ K^–1^,^21^ while Hartree–Fock, MP2, CC2, and DFT with 12 different functionals predict values between +7 and +28 J mol^–1^ K^–1^.^19^ At room temperature, the experimental entropic contribution to *ΔG*^†^ is then −*TΔS*^†^ = +3.6 kcal/mol, while the computational contribution is between −0.5 and −2 kcal/mol. Most of the error persists even after corrections to the harmonic approximation.^19^

Each method also overestimates *ΔH*^†^. The experimental activation enthalpy is 21.1 kcal/mol;^21^ B3LYP-D3/6-311++G** predicts 25.2 kcal/mol, and CC2/aug-cc-pVTZ predicts 29.8 kcal/mol.^19^ CASPT2(10,8)/6-31G* gives *ΔE*^†^ = 31.0 kcal/mol,^22^ which is nearly identical to CC2/aug-cc-pVTZ.^19^ These calculations, like most in the literature, were performed for the inversion TS. In Section S12, we show that highly accurate spin-flip coupled cluster methods yield similar overestimates for the rotational TS.

The errors in *ΔH*^†^ and −*TΔS*^†^ partially cancel for *ΔG*^†^. Indeed, recent work has extensively benchmarked different levels of theory for predicting *ΔG*^†^ of various azoarenes, and found errors near 1 kcal/mol for B3LYP-D3 with some basis sets.^11^ However, given the significant error cancellation for *ΔG*^†^, and the underestimation of *ΔH*^†^ relative to correlated wave function methods, these results should be interpreted with caution.

The second issue concerns the rotational mechanism. While most DFT calculations have been applied to inversion,^11,19,22^ CASPT2 calculations indicate that rotation is in fact preferred for azobenzene.^7^ Yet ref (19) found that the rotational TS cannot be optimized with B3LYP, a finding that we have also reproduced for various derivatives. It is troubling that rotation can be energetically favored, yet the rotational TS cannot be reached through DFT optimization.

### Intersystem Crossing

**Figure 2:**
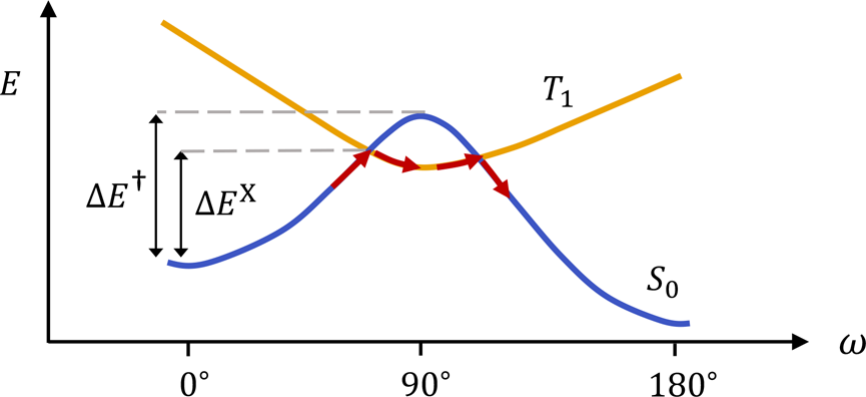
Schematic of triplet-mediated thermal isomerization. Intersystem crossing from singlet (*S*_0_) to triplet (*T*_1_) occurs near ω = 70°. The energy is lower at the crossing point than at the *S*_0_ TS (*ΔE*^X^ < *ΔE*^†^). Recrossing from *T*_1_ to *S*_0_ then occurs near ω = 105°, and isomerization is completed on the *S*_0_ surface.

To address the overestimation of *ΔS*^†^ and *ΔH*^†^ common to all levels of theory, we advocate intersystem crossing (ISC) as the mechanism of thermal isomerization (Figure 2). This was proposed in ref (7) nearly 20 years ago. As discussed below, *ΔG*^†^ is replaced with *ΔG*^X^, the free energy difference at the singlet–triplet crossing geometry X. The rate prefactor *k*_B_*T*/*h* is replaced with the ISC rate *k*_ISC_. Since the crossing geometry has a lower energy than the TS, this approach corrects the overestimation of *ΔH*^†^. Further, the measured *ΔS*^†^ is not a true activation entropy, but in fact related to both log[*k*_ISC_/(*k*_B_*T*/*h*)] and *ΔS*^X^ (eq S28). The negative value of *ΔS*^†^ partly reflects the fact that *k*_ISC_ < *k*_B_*T*/*h*. Computing *k*_ISC_ with CASPT2(14,12)/6-31G* yields good agreement with the effective experimental activation entropy, *ΔS*^eff^, for unsubstituted azobenzene.^7^

We note, however, that isomerization can still proceed through *S*_0_ for certain derivatives and environments, and that the *S*_0_ rate should be compared to the triplet-mediated rate for any new system. Indeed, ref (23) found good agreement between the *S*_0_ CASPT2 rate and the experimental rate for phototrexate in DHFR, though no comparison was made to the experimental activation entropy. For this reason we compute the rate with both Eyring TS theory and ISC for all molecules in this work.

### Multireference Effects

To properly optimize the rotational TS, we show that it is critical to include multireference effects. This is not surprising, since there is a conical intersection when ω is close to 90°.^24^ In this work we use SF-TDDFT,^10^ since it accounts for some double excitations and generally provides an accurate description of conical intersections.^25^ We use the common BHHLYP functional^26^ and 6-31G* basis.^27^ As shown in Section S12, single-reference methods produce rotational TS cusps. This is the likely cause of the failed rotational TS optimizations with standard DFT. Multireference methods, by contrast, produce smooth maxima. We note that the inversion TS is *also* close to a conical intersection,^7^ which further reinforces the need for a multireference treatment of azobenzene TSs.

SF-TDDFT accounts for multireference effects while offering a reasonable balance between cost and accuracy. Empirically it has subcubic scaling,^6^ which makes it far more affordable than highly accurate spin-flip coupled cluster methods that scale as *N*^7^.^28^ Further, as shown in Section S12, SF-TDDFT has similar errors to CASPT2 for rotational barriers in azobenzene. The latter scales as *N*^5^ for a fixed active space, requires manual active space selection, and does not have analytic gradients in most quantum chemistry packages, which are essential for training ML potentials. While SF-TDDFT is not spin-complete, we have found that *S*_0_ spin contamination is rather low. As discussed in Section S8.1, the average square spin in the *S*_0_ state is only 0.16, and we excluded all data with square spin exceeding 1.0. Lastly, the errors in SF-TDDFT are largely systematic, as demonstrated by the strong correlation between predicted and experimental activation free energies in Figure 4. This is discussed further in the Results section below.

### Computational Workflow

Standard quantum chemistry approaches are rather slow. To address this and the above issues, we develop an ISC workflow based on ML potentials^29^ that are trained on multireference SF-TDDFT data.

**Figure 3:**
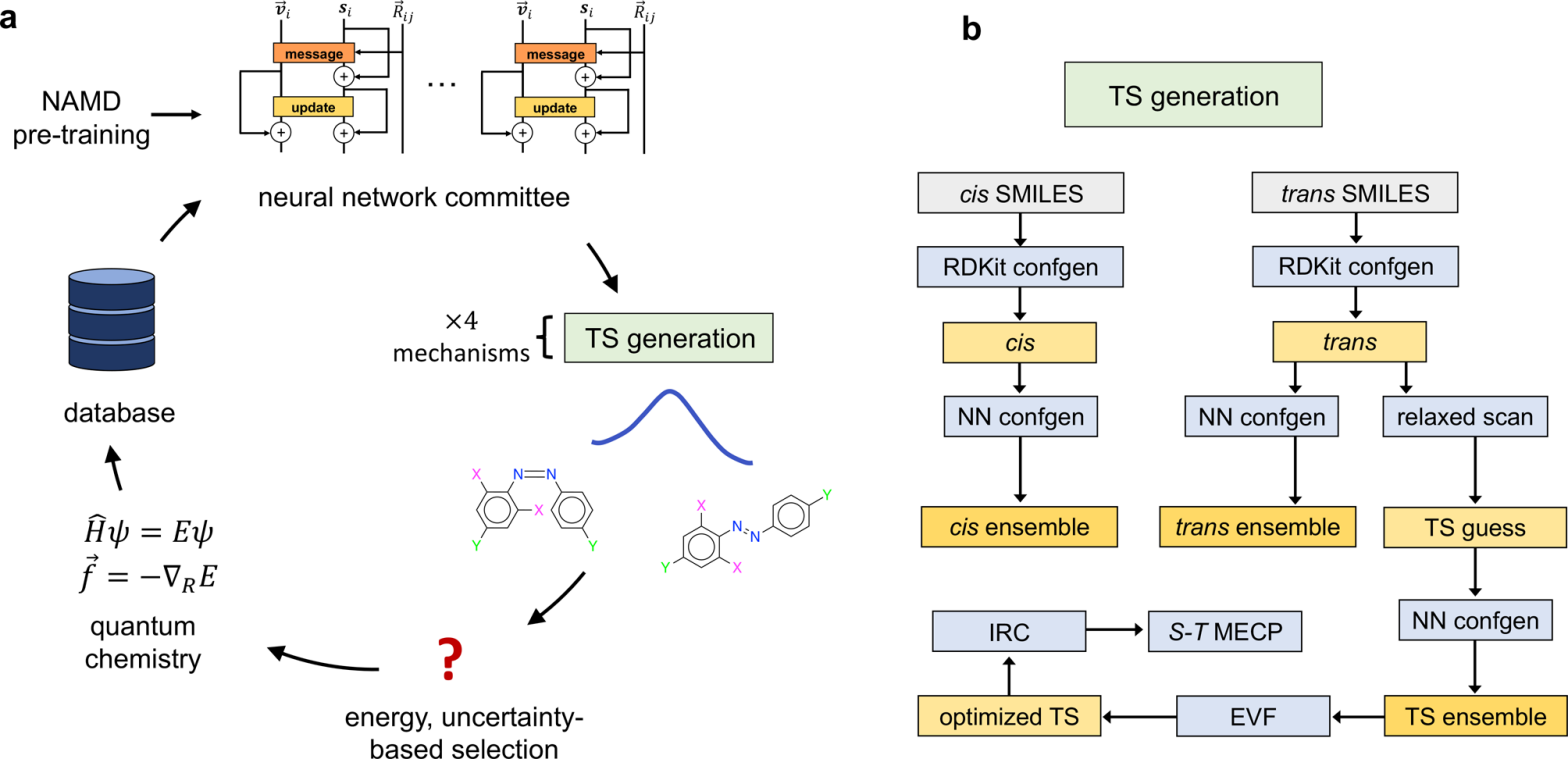
Approach to active learning and TS generation used in this work. (a) Active learning loop for training the NN. (b) Workflow for generating equilibrium and TS geometries. “confgen” stands for conformer generation, which is described in Section S3.2. “*S*–*T* MECP” denotes the search for the minimum-energy singlet–triplet crossings on each side of the TS.

We generate initial TSs through a relaxed scan, and refine the structures with a conformer search^30−32^ and eigenvector following (EVF, Figure 3b). We then use the intrinsic reaction coordinate (IRC)^33^ to locate singlet–triplet crossings on either side of each rotational TS. The geometries are subsequently refined with a minimum energy crossing point (MECP) optimization (Section S3.5). We also apply Eyring TS theory to the TSs and compare the results to the ISC approach.

Four relaxed scans are performed for each of the four mechanisms (two inversion and two rotation). Conformers are generated for each TS guess using a conformational search with fixed CNNC atoms. The five lowest-energy conformers for each mechanism are then optimized with eigenvector following, yielding 20 TSs. The TS with the lowest free energy for each rotational mechanism is used to find the singlet–triplet crossings. ISC does not occur for the inversion mechanism.^7^

Once the workflow is completed, the ISC-based reaction rate is calculated as
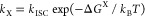
1where *G* is given in eqs S11–S15. *ΔG*^X^ depends on the energies, conformer ensembles, and vibrational frequencies of both the reactant and the crossing geometry. We compute *k*_ISC_ using the approach in ref (34) as described in Section S4. The expression depends on the spin–orbit coupling, temperature, and forces on the singlet and triplet surfaces at the crossing. The spin–orbit coupling is taken as constant, *H*_SO_ ≈ 20 cm^–1^, as described in Section S4. The coupling is about 20 times larger than the typical value for planar aromatic compounds, because of the *n* – π* character of the triplet state.^7^ This corresponds to a 400-fold enhancement in the ISC rate.

We also compute the reaction rate from Eyring TS theory, given by
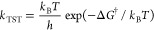
2For ease of comparison we convert ISC-based reaction rates into the form of eq 2, where *ΔG*^†^ is replaced by the effective activation free energy
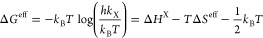
3Δ*S*^eff^ depends on both *k*_ISC_ and *ΔS*^X^, and is given in eq S28.

### ML Models

We train separate models to predict the *S*_0_ energy, *T*_1_ energy, and *S*_0_/*S*_1_ gap. Our models use the PaiNN architecture,^35^ which predicts molecular properties through equivariant message-passing. This approach generates a feature vector for each atom that incorporates information from its surrounding environment. The initial feature vector is generated from the atomic number alone, and is then updated through a set of “messages”. These messages incorporate the distance, orientation, and features of atoms within a cutoff distance. The messages are then used to update the atomic feature vectors. This is performed several times, which leads to information being combined from increasingly distant atoms. Lastly, the atomic features are mapped to per-atom energies using a neural network, which are summed to yield the molecular energy. The forces are computed through automatic differentiation of the energy.

We pretrain the models on 680,736 gas-phase SF/6-31G* calculations from nonadiabatic molecular dynamics (NAMD), which were previously generated in ref (6). We then refine the models using approximately 40,000 SF/6-31G* calculations with a C-PCM model of water.^36−38^ Pretraining on existing gas-phase data means that fewer new solvent calculations are required to reach a target accuracy.^39^

The geometries for SF-TDDFT/C-PCM calculations are generated through active learning^6,39,40^ based on our TS workflow (Figure 3). In each round of active learning, we train three *S*_0_ models on previous SF-TDDFT/C-PCM data (the gas-phase model is used in the first round). The difference in model predictions is used to identify geometries that are poorly described by the model. These high-uncertainty configurations, together with some geometries that are sampled randomly or by energy, receive new quantum chemistry calculations (see Section S1).

## Results

### Model Performance

The model accuracy is shown in Table 1. Mean absolute errors (MAEs) are given for the singlet and triplet models, for both optimized TSs and off-equilibrium geometries sampled during TS conformer generation (see Section S3.2). All geometries come from species outside the training set.

**Table 1:** Model Performance for 334 Species Outside the Training Set^a^

geometry type	model type	singlet *ΔE* error^b^	triplet *ΔE* error^c^	singlet *F⃗* error	triplet *ΔF⃗* error^d^
optimized TS^e^	one model	0.81	0.23	0.44	0.48
	ensemble^f^	0.73	0.19	0.34	0.44
	ensemble, lowest 95% uncertainty^g^	0.66	0.16	0.31	0.42
TS metadynamics^h^	one model	1.09	0.34	0.69	0.55
	ensemble	0.99	0.31	0.54	0.50
	ensemble, lowest 95% uncertainty	0.86	0.27	0.49	0.48

a Units are kcal/mol for energies and kcal/mol/Å for forces. Forces are denoted by *F⃗*.

b Singlet *ΔE* = *E* – *E*_cis_, where *E*_cis_ is the energy of the lowest energy *cis* conformer.

c Triplet *ΔE* = *E*_S_ – *E*_T_, where *E*_S_ is the singlet energy and *E*_T_ is the triplet energy.

d Triplet *ΔF⃗* = *F⃗*_S_ – *F⃗*_T_.

e Four TSs per species, one for each mechanism.

f Three models.

g Uncertainty computed as the standard deviation of the three model predictions.

h Geometries randomly sampled from NN metadynamics for TS conformer generation. Five geometries were sampled for each species.

The model performance is excellent. The error in the barrier energy, *ΔE* = *E*_TS_ – *E*_cis_, is 0.81 kcal/mol for one model and 0.73 kcal/mol for an ensemble of three models. The ensemble error falls to 0.66 kcal/mol after excluding the top 5% most uncertain geometries. These errors are far below 1.0 kcal/mol, which is the typical definition of chemical accuracy. The model error is significantly smaller than the SF-TDDFT error (Section S12).

The force predictions are similarly accurate, with MAEs below 0.45 and 0.7 kcal/(mol Å) for optimized and distorted TSs, respectively. The performance of the triplet model is even better, with errors that are 4 times lower than that of the singlet model. Lastly, the *S*_0_/*S*_1_ gap model has an MAE of 0.68 and 2.35 kcal/mol for optimized *cis* and *trans* geometries, respectively. These are errors of 3.8 and 13.1 nm for a typical absorption wavelength of 400 nm.

### Comparison to Experiment

**Figure 4:**
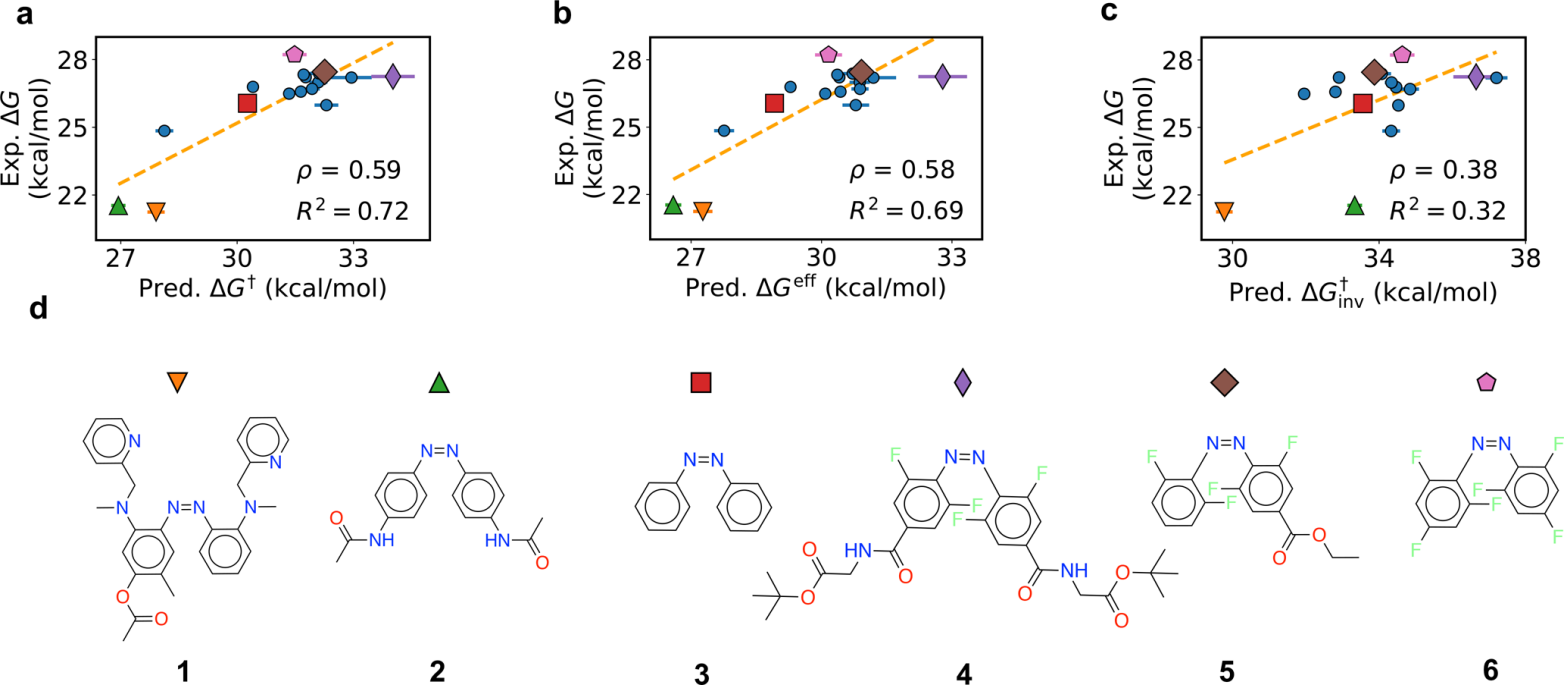
Experimental vs predicted activation free energies. Dotted orange lines are linear regression results from predicted to experimental values. ρ denotes the Spearman rank correlation. *R*^2^ is computed between the regression results and the experimental data. Error bars are the standard deviation of the energy predictions from three models. (a) Prediction accuracy using TS theory. (b) Prediction accuracy using intersystem crossing. (c) As in panel a, but with only the inversion mechanism. (d) Selected compounds highlighted in panels a–c.

The predicted and experimental activation free energies are compared in Figure 4. The experimental data come from refs (17, 18, 20, and 41−47) and can be found in the file containing the virtual screening results. Twenty-six measurements were accessed in total, and 17 of these were used in Figure 4. We only used the measurements performed in solvent with dielectric constant ε ≥ 30. Our model was trained on implicit solvent calculations using ε = 78.4 for water. However, we found that the quantum chemistry energies were quite similar when using ε = 30, and so included these measurements in the benchmark as well.

The results of TS and ISC theory are shown in Figure 4a,b, respectively. The rotation mechanism was favored in TS theory for all compounds shown, and so Figure 4a equivalently shows *ΔG*^†^ from rotation. Figure 4c shows the results of TS theory when considering only the two inversion mechanisms.

The correlation with experiment is quite good for both TS and ISC theory. Both have Spearman rank coefficients ρ near 0.6, and both have *R*^2^ near 0.7 after linear regression. The MAEs after regression are 0.79 and 0.85 kcal/mol for TS and ISC theory, respectively. When including all 26 measurements performed in any solvent, we find that ρ actually increases to 0.67 for both methods. *R*^2^ falls to 0.58 and 0.63 for TS and ISC theory, respectively. The respective MAEs climb to 0.92 and 0.97 kcal/mol.

The species are properly separated into low-, medium-, and high-barrier groups in Figure 4. For example, species **1** and **2** are predicted to have low barriers and the fluorinated derivatives **4**–**6** to have high barriers, and azobenzene is predicted to lie in the middle. The models even have some success comparing fluorinated derivatives to each other, with ρ = 0.33 and 0.42 among these species for *ΔG*^†^ and *ΔG*^eff^, respectively. However, the two approaches give *R*^2^ = 0.02 and 0.04, respectively. This means that the numerical error is close to that of a random predictor, even though the rankings are better than random.

Both approaches overestimate the barriers, on average by 4.94 and 3.76 kcal/mol for TS theory and ISC, respectively. However, the overestimation is largely systematic, as demonstrated by the high *R*^2^ value after linear regression. Further, it is a consequence of SF-TDDFT, not the models. Indeed, as shown in Section S12, *ΔH* is well-reproduced by the accurate and expensive method SF-EOM-CCSD(dT).^28^ SF-TDDFT overestimates *ΔH* with respect to both experiment and SF-EOM-CCSD(dT). Transfer learning to this higher level of theory could therefore be of interest in the future.

*ΔG*^eff^ is lower than *ΔG*^†^ on average, but otherwise their trends are quite similar. One reason is that rotation is the predicted mechanism for all species. Since each singlet–triplet crossing is on either side of a rotational TS, its energy is correlated with that of the TS. Indeed, the correlation between the two methods is near unity, with ρ = 0.97 and *R*^2^ = 0.98. This reflects the fact that *E*_rot_^†^ – *E*^X^ and *k*_ISC_ are nearly constant among different species. However, we explain below that noticeable differences arise when screening large virtual libraries.

The approaches’ strong performance should be contrasted with TS theory using only inversion. These results are shown in Figure 4c. The performance is far worse than using rotation or ISC, with *R*^2^ reduced by over 50%, and ρ reduced by over 35%. The MAEs after regression are 1.24 kcal/mol for polar solvents and 1.43 kcal/mol for all data. Note that most works with DFT have only produced inversion TSs. This is likely because of failed rotation optimizations, which we attribute to the single-reference nature of DFT and the associated TS cusps (Section S12). Similar difficulties were found in ref (9). Our results highlight the importance of rotation and multireference effects.

Note that all species in Figure 4 were in the training set. Hence the comparison to experiment does not measure the model’s ability to generalize to new compounds. Rather, it mainly measures the reliability of the workflow and the underlying quantum chemistry. The models’ ability to generalize to new species is shown in Table 1.

### Virtual Screening

With reliable models and predictive workflows, we next screened a virtual library of azobenzene derivatives for key properties in photopharmacology. We ran the workflow of Figure 3b for 25,000 compounds in all. The compounds were generated using the common literature substitution patterns in Figure 7d, following the approach of refs (6 and 48). The substituents are a combination of literature groups and basic chemical moieties, and can be found with the screening results online. After applying various filters, such as the right number of imaginary frequencies, converged TSs for all mechanisms, and the proper IRC end points, we were left with 19,000 species in total (see Section S9).

#### Distributions

**Figure 5:**
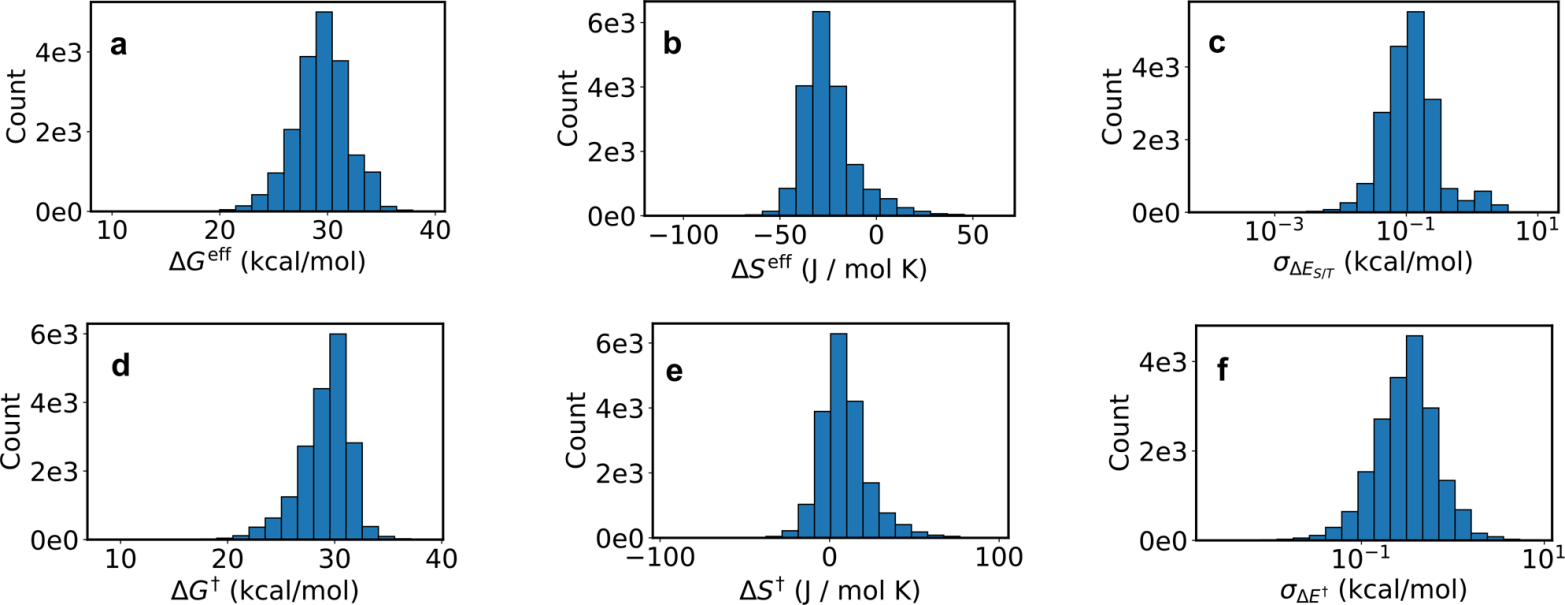
Distribution of various quantities among the 19,000 screened derivatives. (a) Effective activation free energy. (b) Effective activation entropy. (c) Model uncertainty in the singlet–triplet gap at MECPs. (d, e) As in panels a and b, but using TS theory. (f) Model uncertainty in the activation energies.

Figure 5a shows the distribution of *ΔG*^eff^ from this screen. The mean and median are 29.6 and 29.7 kcal/mol, respectively, while unsubstituted azobenzene has a value of 28.9 kcal/mol. The average derivative is thus more kinetically stable than azobenzene. The standard deviation is 2.6 kcal/mol, which is a factor of 80 in the isomerization rate. 39% of species (7,400) have a lifetime that is over 10× that of azobenzene; 19% (3,600) have a lifetime that is less than 1/10th. We conclude that the lifetime is highly tunable using the substitutions in this work.

Figure 5b shows the effective activation entropy. The mean is *–*24.5 J/(mol K), and the median is *–*26.9 J/(mol K). The calculated and experimental values for azobenzene are *–*29.0 J/(mol K) and *–*50.2 J/(mol K),^21^ respectively. The associated error in the entropic free energy is 1.5 kcal/mol. Using the TS approach gives *ΔS*^†^ = 4.7 J/(mol K), which has a much higher entropic free energy error of 3.9 kcal/mol. Few other derivatives have experimental activation entropies, and of those that do, most have values near that of azobenzene. However, of the few with values far from azobenzene, none were predicted accurately by the model.^20,47,49^ These cases should be investigated in more detail in the future.

Figure 5d,e is analogous to Figure 5a,b, but with TS theory instead of the ISC approach. Each distribution resembles its partner from ISC theory. However, *ΔG*^†^ is more asymmetric than *ΔG*^eff^, with a much steeper drop-off for higher barriers. The reason is as follows. Within TS theory using SF-TDDFT in water, rotation is more often the preferred mechanism (Section S6). However, inversion can become preferred for species with high enough rotation barriers. This mechanism is not available in ISC theory, since *T*_1_ is always higher than *S*_0_ during inversion. Hence inversion lowers the high barriers in TS theory, but cannot do the same in ISC theory.

In principle one should calculate both *ΔG*^†^ and *ΔG*^eff^, and use the lower value for the reaction rate. If *ΔG*_inv_^†^ were low enough, it would replace *ΔG*^eff^ in the high barrier regime, and so the steep drop-off of Figure 5d would be observed. However, as discussed in Section S6, SF-TDDFT is not accurate enough to compare absolute *ΔG*^†^ and *ΔG*^eff^ directly. Hence a more accurate treatment of this problem would be of interest in the future.

Figure 5c,f shows the model uncertainty in the MECP singlet–triplet gap and the activation energy, respectively. Both are quite low, indicating high model confidence for the derivatives studied here. The mean uncertainties are 0.21 and 0.41 kcal/mol for the singlet–triplet gap and activation energy, respectively. This is consistent with the error trends in Table 1. The uncertainty should be interpreted with caution, however, as neural network ensembles tend to be overconfident.^50^ Large uncertainty necessarily means high error, as demonstrated by the error reduction in Table 1 when excluding the most uncertain geometries; however, low uncertainty does not guarantee low error. A more detailed examination of uncertainty, including calibration to the observed error and use of different architectures in the ensemble, is left to future work.

#### Targeting Desired Properties

Absorption wavelength and thermal stability are two key properties in the design of photoactive drugs. The preferred absorption range is generally 650–900 nm, since human tissue is transparent only in this narrow region of the near-IR.^4^ For photoactive drugs one typically wants the isomerization barrier to be as high as possible, so that the unstable isomer is active for as long as possible. For ion channel blockers, by contrast, the target lifetime is usually milliseconds.^51^ For reference, the half-life of azobenzene is 1.4 days in benzene solution at 35 °C.^100^ Here we use the screening results to identify red-shifted derivatives with high or low barriers.

**Figure 6:**
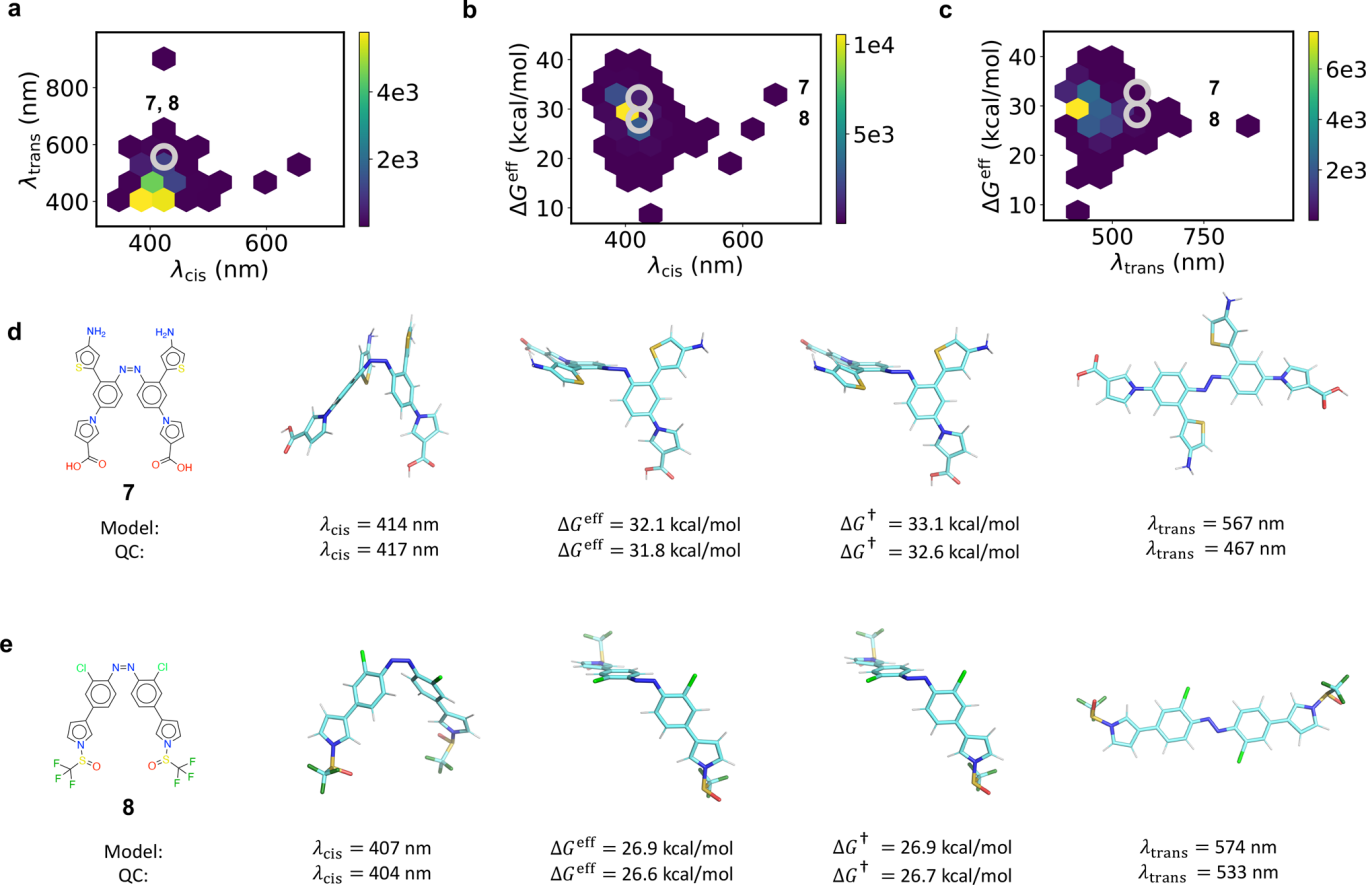
Absorption wavelengths and thermal barriers. (a) *Trans* vs *cis* and absorption wavelengths. (b, c) *ΔG*^eff^ vs *cis* and *trans* absorption wavelengths, respectively. (d, e) The two compounds of interest. For each panel, the graph is shown on the left, followed by the *cis* geometry, the singlet–triplet crossing closer to *cis*, the TS, and the *trans* geometry. Model and quantum chemistry (QC) predictions are shown below. The compounds are circled in panels a–c.

Figure 6 shows combinations of λ_cis_, λ_trans_, and *ΔG*^eff^, where λ_*i*_ is the absorption wavelength of isomer *i*. We found that *trans* is the more stable isomer for 99.6% of all species. The stable isomer is usually the one activated by light, and so λ_trans_ is usually the quantity of interest. Figure 6a,c shows that red-shifting to the near-IR is quite difficult. Out of 19,000 compounds, only five have λ_trans_ > 600 nm. Of these, only one has a barrier greater than that of azobenzene. There are 1,641 species with λ_trans_ > 500 nm (8.7%), including 475 with a barrier greater than azobenzene (2.5%). However, the majority of predictions over 500 nm are significant overestimates. As discussed below, most are actually closer to 470 nm.

Two red-shifted species are shown in Figure 6d,e. Compound **7** has a high barrier, and compound **8** has a low barrier (top and bottom, respectively). We confirmed these predictions using single-point SF-TDDFT calculations. The quantum chemical activation free energies are shown below the model predictions, and the two agree quite well. We used the model results for the quasiharmonic and conformational contributions to the enthalpy and entropy, since a numerical Hessian with SF-TDDFT would be prohibitively expensive.

While the barriers agree quite well with SF-TDDFT, the *trans* absorption wavelengths are overestimated. For example, the predicted and true wavelengths are 567 and 467 nm for compound **7**, and 574 and 533 nm for compound **8**. Moreover, this latter result is somewhat suspect, since the first excited state with SF-TDDFT has square spin ⟨*S*^2^⟩ = 1.2, indicating high spin contamination. Indeed, a restricted TDDFT calculation with the ωB97X-D3 functional^52^ and the def2-SVP basis^53^ yielded λ_trans_ = 470 nm. These results are common: after performing calculations for the 40 species with the highest *trans* absorption wavelengths, we found that all either had true values around 470 nm or had significant spin contamination leading to untrustworthy results.

We note that while the *S*_1_ spin contamination was severe for some species with ultrahigh absorption wavelengths, it was otherwise low in general. Indeed, the average square spin of the *S*_1_ state was 0.37 in the training set. This is higher than the mean value of 0.16 for the *S*_0_ state, but still reasonable. The maximum ⟨*S*^2^⟩ allowed in the training set was 1.5 for the *S*_1_ state. To avoid any *S*_1_ spin contamination, one could always fine-tune the model with a small data set of excitation energies from spin-complete TDDFT and ωB97X-D3. Multireference effects would likely be small for equilibrium structures, and only a few new calculations would be needed for fine-tuning.^6,39^

These results lead to several conclusions. First, red-shifting is quite difficult. This is shown by the fact that most model predictions above 500 nm are actually error outliers. The associated wavelengths are still much higher than the base compound, but quite far from the predicted values. Second, we find that spin contamination is severe for many of the high-λ predictions. Third, on a positive note, the model is able to identify red-shifted species with either high or low barriers, even though the red-shift is overestimated. This is encouraging for virtual screening in photopharmacology.

The quantum chemistry absorption wavelengths also come with several sources of uncertainty. They include errors in SF-TDDFT, implicit treatment of the solvent, and use of static structures instead of thermally sampled geometries.^6^ Moreover, since the experimental absorption width is usually quite large, compounds can often absorb at wavelengths 100–200 nm higher than their peak.^54^

#### Graph–Property Relationships

Here we analyze the relationship between substitution and chemical properties. Observing and explaining general trends will enable more focused candidate screening in the future. Previous papers have also explored these relationships computationally;^12,17,18,20,55^ we build on their conclusions and extend them to other substitution patterns and groups.

**Figure 7:**
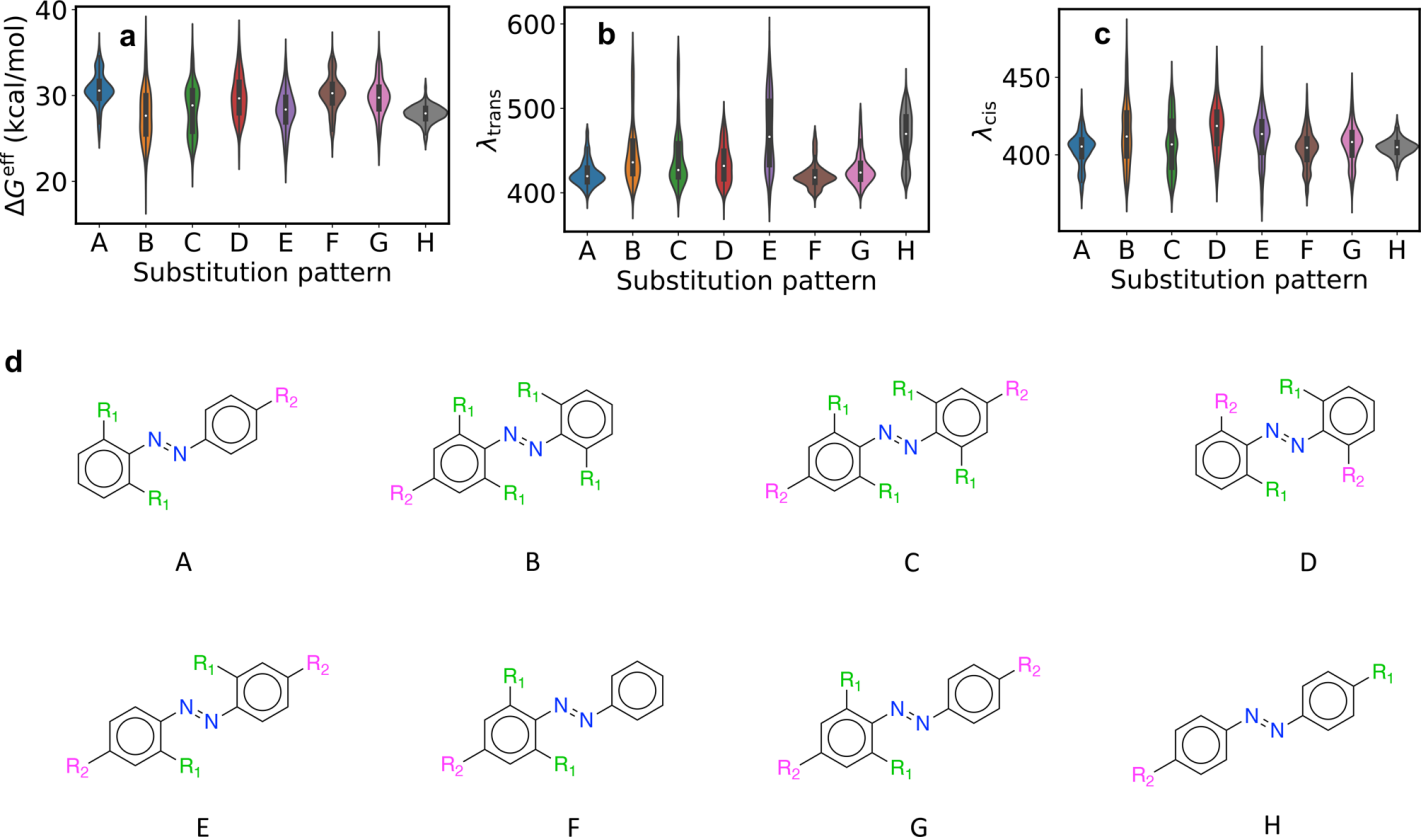
Chemical properties by substitution pattern. 3-σ outliers were removed for ease of visualization. (a) Effective activation free energy. (b) *trans* absorption wavelength. (c) *cis* absorption wavelength. (d) Substitution patterns.

Figure 7 shows barrier heights and absorption wavelengths by substitution pattern. Figure 7a shows that motifs A, F, and G have bimodal *ΔG*^eff^ distributions, with high barriers around the second mode. This is explored more below. Patterns B, C, and E have below-average barriers and elongated distributions, while pattern H has a tight distribution and the lowest mean barrier.

Figure 7b,c shows λ_trans_ and λ_cis_, respectively. Intriguingly, we see that the distributions of classes E and H are very elongated for λ_trans_. The same is true to a lesser extent for B and C. While patterns like A, D, and F are tightly concentrated between 400 and 425 nm, class E samples from 400 to 525 nm with almost equal probability. The λ_cis_ distributions are much tighter.

**Figure 8:**
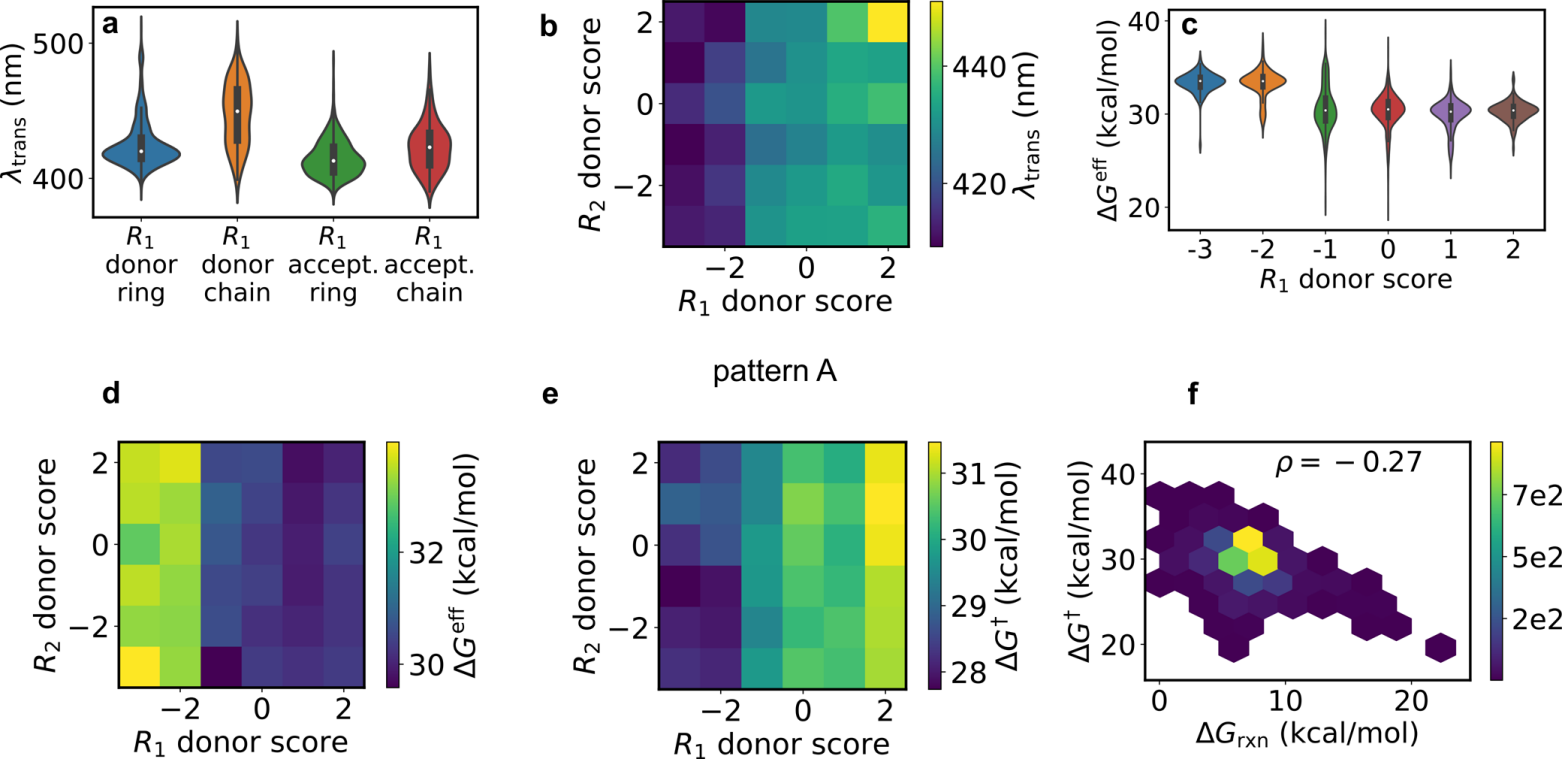
Relationships among substituent properties, free energies, and absorption wavelengths for pattern A. The pattern is shown in Figure 7d. (a) λ_trans_ for donor/acceptor and ring/nonring *R*_1_ substituents. (b) λ_trans_ as a function of *R*_1_ and *R*_2_ donor scores. (c) *ΔG*^eff^ as a function of *R*_1_ donor score. (d) *ΔG*^eff^ as a function of both *R*_1_ and *R*_2_ donor scores. (e) As in panel d, but for *ΔG*^†^. (f) *ΔG*^eff^ vs *ΔG*_rxn_.

To better understand these results, we next analyze the relationship between substituent properties and molecule properties for pattern A. Figure 8a shows that λ_trans_ is maximized when *R*_1_ is a nonring donor. In particular, Figure 8b shows that this occurs when both *R*_1_ and *R*_2_ are strong donors. Each box in this panel shows the root-mean-square of λ_trans_ for the given (*R*_1_, *R*_2_) pair, computed as min{λ_trans_} + mean{(λ_trans_ – min{λ_trans_})^2^}^0.5^. This gives a mean that is weighted toward higher values, reflecting our interest in maximizing λ_trans_ better than a simple mean. The results are somewhat unexpected: a simple picture of donors raising the HOMO and acceptors lowering the LUMO would predict a red-shift for strong donors *or* acceptors. Yet the actual effect is only noticeable for donors.

Figure 8c shows that strong *R*_1_ acceptors lead to very high barriers, with narrow distributions centered around 33 kcal/mol. This is consistent with ref (18), which reported long thermal lifetimes for ortho-fluoro-substituted azobenzenes. Donor-substituted azobenzenes have noticeably lower barriers. However, the associated barriers are tightly concentrated near 30 kcal/mol, which is higher than that of unsubstituted azobenzene. This is encouraging, since it means that substitution with two donors can red-shift the absorption wavelength without decreasing the barrier.

Figure 8d reinforces that *ΔG*^eff^ has the opposite trend of λ_trans_ (in this plot we use the mean for each box). Again, however, we see that even the lowest barriers in the upper right corner are similar to that of azobenzene. Intriguingly, Figure 8e shows the opposite trend for *ΔG*^†^. This may be related to the absence of an inversion mechanism for the ISC approach. This result reinforces the need for high-accuracy quantum chemistry to accurately compare *ΔG*^†^ and *ΔG*^eff^.

Figure 8f shows the relationship between the activation free energy and the reaction free energy *ΔG*_rxn_, where *ΔG*_rxn_ = *G*_cis_ – *G*_trans_. The Bell–Evans–Polanyi principle^56,57^ states that the reaction enthalpy and activation enthalpy are linearly related for reactions in the same family. This relationship was also tested for azobenzene derivatives in ref (17). We see a moderate negative correlation between the two quantities, with Spearman ρ equal to −0.27. Hence *ΔG*^eff^ can be increased by making the *cis* isomer more stable. However, the modest correlation means that this is not the full story, and that the TS and MECP energies must be explicitly considered.

## Discussion

The main source of error in our approach is the underlying quantum chemistry calculations. As discussed in Section S12, expensive wave function methods give lower barriers than SF-TDDFT, but still give different answers from each other. On balance the most accurate method seems to be SF-EOM-CCSD(dT), but its prohibitive *N*^7^ scaling^28^ makes it a poor candidate for transfer learning. Future work should focus on accurate quantum chemical approaches that do not need manual setup, such as selection of active spaces, and that are affordable enough for transfer learning.

Another limitation is that we have not considered azobenzene protonation and azo-hydrazone tautomerism. These effects can be facilitated by substituents such as NH_2_ and OH, and by solvation in a protic solvent, weakening the N=N double bond and lowering the isomerization barrier.^58−60^ Protonation from the solvent is not accounted for in a PCM description. Incorporating automated protonation tools^61^ into our workflow would be of interest in the future. Similarly, the protein environment for a given target in photopharmacology can also affect the isomerization rate.^23,62^ Incorporating these effects for a specific target, as in ref (23), is of interest for future work.

From the perspective of property optimization, the biggest remaining challenge is red-shifting. While it is straightforward to reach λ = 470 nm for *trans* isomers, it appears very difficult to reach λ = 550–600 nm. Averaging the gap over thermally sampled geometries may increase the wavelength and improve prediction accuracy.^6^ Including bulky groups in all four ortho positions may also increase the wavelength,^6^ but at the potential cost of synthetic accessibility. A more targeted approach to wavelength optimization could be of interest in the future. For example, methods such as Monte Carlo tree search^63^ could likely improve over virtual screening of combinatorial libraries.

## Conclusions

We have presented a fast and automated method for predicting the isomerization barriers of azobenzene derivatives. The approach can compute the activation free energy through TS theory or ISC theory. We have demonstrated the accuracy of the underlying ML model with respect to SF-TDDFT, reproduced trends in the experimental isomerization rate, and argued for rotation-based ISC as the reaction mechanism. Our software is fast, accurate, and easily accessible to the community, making it a valuable tool for computational design of photoactive molecules. Future work will focus on more accurate quantum chemistry methods and more targeted molecular generation.

## Supporting Information Available

Extended methods, details of molecule generation, details of optimization, nonadiabatic transition state theory, activation free energies by mechanism, thermal isomerization rates, details of training, filtering protocol in screening, collection of experimental data, note on mechanisms, and quantum chemistry benchmark (PDF)
