# Supplementary material for: Thermal Half-Lives of Azobenzene Derivatives: Virtual Screening Based on Intersystem Crossing Using a Machine Learning Potential
Source: ACS Cent Sci. 2023 Jan 25;9(2):166–76. doi: 10.1021/acscentsci.2c00897 (PMC9951306; doi:10.1021/acscentsci.2c00897)
Supplement: Supplementary file 1 — oc2c00897_si_001.pdf [file oc2c00897_si_001.pdf]

Supporting Information
Thermal half-lives of azobenzene derivatives: virtual screening based on intersystem crossing using a machine learning potential

Simon Axelrod,[†,‡] Eugene Shakhnovich,[†] and Rafael Gómez-Bombarelli[*,‡]

†*Department of Chemistry and Chemical Biology, Harvard University, Cambridge, MA, 02138*
‡*Department of Materials Science and Engineering, Massachusetts Institute of Technology, Cambridge, MA, 02139*

E-mail: rafagb@mit.edu

S1 Extended methods

All models used the PaiNN architecture[1] and were implemented in PyTorch[2]. As in our previous work, we used five convolutions instead of the three used originally, as this substantially improved model performance[3]. We also allowed the k values in the radial basis functions to be updated during training. Remaining hyperparameters can be found in Ref.[3], and an in-depth explanation of their meaning can be found in Ref.[1]. Further hyperparameter optimization is likely possible[4].

After training the model on data without empirical dispersion, we added D3BJ dispersion[5,6] to the total energy prediction:

$$E = E_{\mathrm{model}} + E_{\mathrm{D3}} \tag{S1}$$

$$\mathbf{F} = -\nabla E_{\mathrm{model}} - \nabla E_{\mathrm{D3}} \tag{S2}$$

The forces were computed with automatic differentation[7]. Dispersion parameters were taken from the PhysNet repository[8,9]. No cutoff was used. D3 dispersion is a function only of the atom types and positions, and could therefore be added analytically without approximation. The model performance in the main text used SF-D3BJ TDDFT as the ground truth.

The geometries for SF-TDDFT/C-PCM calculations were generated through active learning (Fig. 3(a)). In each round of active learning, the TS workflow in Fig. 3(b) was performed for 1,000 (SMILES, mechanism) pairs using one of the trained models. The pairs were randomly sampled from a set of 73,000 reactions with four mechanisms each. We selected 3,000 generated geometries for DFT calculations, added the new data to the training set, and repeated. 50% of the geometries were selected by uncertainty, 30% by energy, and 20% randomly. For uncertainty-based selection, we chose the geometries with the highest variance in forces predicted by the three models. For energy selection, we sampled a geometry i with probability $p_i \propto e^{E_i/k_{\mathrm{B}}T}$. The energy was computed relative to other geometries of the same species in the same simulation. The energy selection sampled near-TS geometries from relaxed scans, and high-energy MTD structures from conformer generation.

Active learning was repeated 15 times, yielding 43,938 calculations in total. The final model was trained on 42,938 geometries, with 500 used for validation and 500 for testing.

S2 Molecule generation

Molecules were generated using the patterns in Fig. 7(d). These are common substitution patterns in the literature; see, for example, the synthesized species in Refs.[10–19]. Some of the functional groups were common azobenzene substituents aggregated in Ref.[3], with new groups added from Refs.[20–22]. Others were basic chemical moieties, such as benzene, ethane, $\mathrm{R-CF_3}$, and so on.

We aimed to avoid steric clashes in tetra-ortho substitution, since this can make experimental synthesis more difficult. For this reason we separated substituents by size. Those with four total atoms or fewer were labeled small,

and all others were labeled large. For all tetra-ortho substitutions, we only allowed one side of the N=N bond to be substituted with large groups. The other side could only be substituted with small groups. Substitution with small groups at all positions was also allowed.

S3 Optimizations

S3.1 Relaxed scans

To perform the relaxed scans, we first identified the CNNC atoms using a substructure search of azobenzene in RDKit[23]. We then adjusted their angles and/or dihedral angle by a constant amount in each step of the scan. The angles and dihedrals were adjusted using the atomic simulation environment (ASE)[24]. For the inversion mechanism we set the final CNN or NNC angle to 179.5°. We used 179.5° instead of 180° because the Cartesian derivative of the latter is undefined, and hence the constraining forces described below would also be undefined. For rotation we set the final dihedral angle to ±90°. We also set the final CNN and NNC angles to 122°. Without this constraint, we found that some of the scans collapsed to inversion TSs instead of rotation TSs. 122° is the angle of the rotational TS when optimizing azobenzene with SF-TDDFT[25].

20 steps were used in each scan. After the angles and/or dihedrals were adjusted, the geometry was optimized with forces given by $\mathbf{F} + \mathbf{F}_{\text{restrain}}$. The restraining forces are the negative gradients of the following restraining energies:

$$E_{\alpha,n} = k_\alpha(\alpha - \alpha_n)^2 \tag{S3}$$

$$E_{\omega,n} = -k_\omega \cos(\omega - \omega_n). \tag{S4}$$

Here α denotes an angle, ω denotes a dihedral, n denotes the nth step of the optimization, and α_n, ω_n are target values at the nth step. E_α was used for the inversion mechanism, while both E_α and E_ω were used for the rotation mechanism.

k_α and k_ω were each set to 1 Ha. The optimization was performed with the BFGS algorithm[26–29] in ASE. A rather loose convergence threshold of $f_{\text{max}} = 0.05$ eV/Å was used. This was because of the conformer generation performed after the scans. As described in Sec. S3.2, each stage of conformer generation was restarted if a new conformer had a lower energy than the seed conformer. We used a tolerance of $f_{\text{max}} = 0.05$ eV/Å to minimize the optimization time spent on high-energy conformers. Tight convergence criteria were only used once no lower energy conformers were found. If the seed conformer were optimized with tighter thresholds than the generated ones, it could have a lower energy simply due to its stricter thresholds. Hence it needed to be optimized with the same thresholds.

Angles and dihedrals were set with ASE at each step. ASE adjusts only these internal coordinates, without rotating the rest of the molecule as a solid body. RDKit uses a solid body rotation, and in principle should then require fewer optimization steps at each stage of the scan. However, we found that this led to unstable scans, and so we used ASE instead.

For all optimizations and dynamic simulations we updated the neighbor list every ten steps. To account for neighbors coming into the cutoff radius between updates, we computed the neighbors using a cutoff of $r_{\text{cut}} + r_{\text{skin}}$, where $r_{\text{cut}} = 5.0$ Å is the model cutoff radius and $r_{\text{skin}} = 2.0$ Å is the cutoff skin. Distances between atoms and neighbors were computed at each step, and only those within r_{cut} of each other were used in the model.

S3.2 Conformer generation

Conformer searches were performed for all reactants, products, and transition states. We implemented a method based on CREST[31] using our NN. CREST combines metadynamics, high-temperature molecular dynamics, and genetic structure crossing[33] to sample conformational space. The sampled geometries are then optimized with a multi-level filtering scheme. In this approach, optimizations are performed with progressively tighter thresholds, and structures are discarded if their energy is above a maximum value that is lowered in each step. By default CREST uses the fast semi-empirical method GFN2-xTB[34] to compute energies and forces.

The key component for phase space exploration is metadynamics (MTD). The collective variables used for MTD are the root-mean-square displacements (RMSDs) from previously visited structures. This drives the molecule away from previously visited configurations and towards new regions of phase space. The associated biasing potential is given by

$$V_{\text{bias}} = \sum_i^n k_i \exp(-\alpha_i \Delta_i^2). \tag{S5}$$

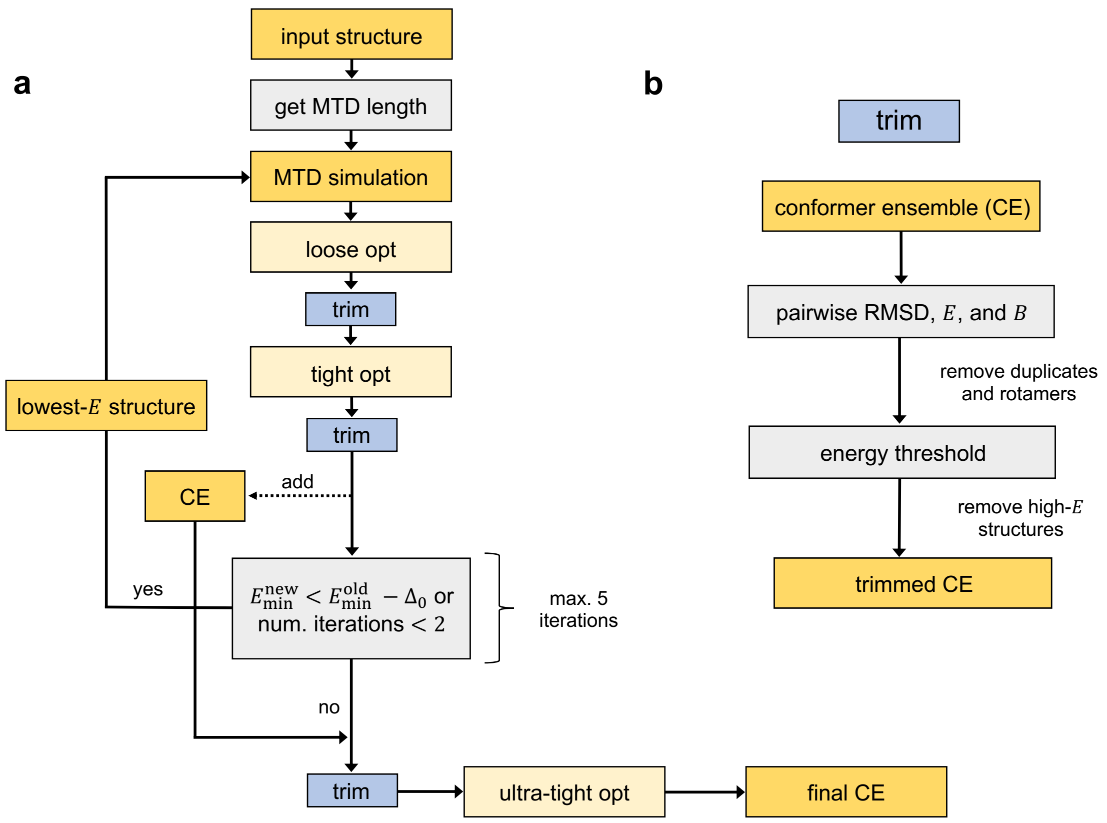

Figure S1: Outline of the conformer generation workflow used in this work. (a) Overview of the approach. (b) Details of the trimming procedure.

Here the sum is over n previously visited structures, where a new structure is added every 1 ps. Δ_i is the RMSD of the current structure with respect to the ith previous structure, k_i is a pushing strength parameter, and α_i is a width parameter. The forces at each step are the sum of the actual forces and the negative gradient of V_{bias}. 14 MTD runs are performed in CREST, using different combinations of α_i and k_i.

Initially we used CREST to perform conformer searches. However, given the speed of relaxed scans and eigenvector following with our NN, we found that CREST was by far the most time-consuming step in the workflow. For example, with access to 24 Xeon-P8 CPU nodes and 1,152 cores in total, we ran 82 CREST jobs at a time with 14 cores per job. The average job took 4.2 hours, which meant a throughput of 470 geometries per day. Given four TS guesses per species, plus the reactant and product geometries, this meant that only 78 species could be screened per day. Screening 25,000 species, as done in this work, would have taken 11 months.

CREST has built-in options for conformer searches that are faster but less extensive. These involve fewer MTD runs and tighter energy windows for each level of optimization. However, given less extensive sampling and tighter energy windows, it makes sense to use our NN instead xTB. For example, CREST has a default energy window of 6.0 kcal/mol for the final ensemble, even though conformers of energy greater than approximately 2.5 kcal/mol are not populated at room temperature. This is to compensate for errors in xTB, so that low-lying conformers mistakenly assigned a high energy by xTB are not discarded. These conformers can be subsequently re-ranked or re-optimized with a higher level of theory. Therefore, it is more reasonable to use a tight energy window for the NN, which is specifically trained to reproduce SF-TDDFT for azobenzene derivatives. Similarly, the sampling should be more extensive for xTB than for the NN. This is because errors in xTB mean that conformational space must be thoroughly sampled up to an energy of 6.0 kcal/mol. The NN, by contrast, only needs to thoroughly sample the space up to an energy of 2.5 kcal/mol. Alternatively, one could retain the extensive sampling but use the ultra-fast GFN force-field[35]. However, we found that the force-field produced poor results for TSs.

We therefore implemented our own conformer search with the NN, and used tighter energy windows and fewer dynamics simulations. The workflow is shown schematically in Fig. S1. The approach closely follows that of CREST, but uses only one MTD run per stage, and does not use MD or genetic structure crossing. As in CREST, we determined the MTD time using a flexibility measure derived from the chemical graph (the current formula in

Table S1: Parameters used in our NN conformer generation workflow. From top to bottom, the sections contain MTD parameters, optimization parameters, and `cregen` parameters. N is the number of atoms in the system. The `cregen` parameters are the defaults used in CENSO, a program that refines CREST ensembles with DFT[32].

Parameter	Meaning	Value
t_{new}	time between adding reference structures to V_{bias}	1 ps
κ	turn-on parameter for adding new MTD Gaussians[30]	0.03
k'	$k = Nk'$ is the biasing strength in Eq. (S5)[31]	1.5 mE_{h}
α	Gaussian width in Eq. (S5)	0.5 Bohr^{-2}
Δt	time step	2 fs
m_{H}	hydrogen mass used in dynamics	4 amu
thermostat	method for enforcing average temperature during dynamics	Nosé-Hoover
Q	Nosé-Hoover effective mass	$(3N-6)\cdot\tau^2 k_{\mathrm{B}}T$
τ	Nosé-Hoover relaxation time	100 fs
T	simulation temperature	298.15 K
Δ_0	see Fig. S1(a)	0.2 kcal/mol
$f_{\mathrm{max}}^{\mathrm{loose}}$	maximum force component in loose opt	0.2 eV/Å
$f_{\mathrm{max}}^{\mathrm{tight}}$	maximum force component in tight opt	0.05 eV/Å
$f_{\mathrm{max}}^{\mathrm{ultra}}$	maximum force component in ultra-tight opt	0.005 eV/Å
$E_{\mathrm{win}}^{\mathrm{loose}}$	energy window for retaining conformers after loose opt	15.0 kcal/mol
$E_{\mathrm{win}}^{\mathrm{tight}}$	energy window for retaining conformers after tight opt	8.0 kcal/mol
$E_{\mathrm{win}}^{\mathrm{ultra}}$	energy window for retaining conformers after ultra-tight opt	2.5 kcal/mol
ΔE_{thr}	two geometries cannot be duplicates or rotamers if their energies differ by more than ΔE_{thr}	0.15 kcal/mol
ΔR_{thr}	two geometries cannot be duplicates if their RMSD is more than ΔR_{thr}	0.175 Å
ΔB_{thr}	two geometries cannot be rotamers if their rotational constants differ by more than ΔB_{thr}	0.03 (3%)

CREST differs from that of the original publication; we used the up-to-date version in the source code[36], commit 6bb6355). To partially compensate for the decreased total simulation time, we used a minimum MTD time of 15 ps, instead of the 5 ps used in CREST. The parameters used in our workflow are given in Table S1.

Several points require further discussion. First, we used the CREST `cregen` tool to remove both duplicates and rotamers in the ensemble. Duplicates are pairs of geometries with $\Delta E < \Delta E_{\mathrm{thr}}$, RMSD $< R_{\mathrm{thr}}$, and $B < B_{\mathrm{thr}}$, where Δ is the difference between the two quantities, E is the energy, B is the rotational constant, and thr denotes a threshold. Rotamers are structures with $\Delta E < \Delta E_{\mathrm{thr}}$, RMSD $> R_{\mathrm{thr}}$, and $B < B_{\mathrm{thr}}$. We removed rotamers because the difference in rotamer count per conformer should be small, and because accounting for all rotamers is quite difficult. This is especially true when MD and genetic structure crossing are not included, since they are included in CREST primarily to find rotamers[31].

Second, we used the L-BFGS algorithm[37] in ASE to perform optimizations. The convergence criterion is $\max_{i,\alpha} |f_{i,\alpha}| < f_{\mathrm{max}}$, where $f_{i,\alpha}$ is a force component, $i \in \{1, N\}$ is the atom index, $\alpha \in \{x, y, z\}$ is the Cartesian index, and f_{max} is a threshold. For equilibrium geometries we used $f_{\mathrm{max}}^{\mathrm{ultra}} = 0.005$ eV/Å. For TS geometries we used $f_{\mathrm{max}}^{\mathrm{ultra}} = 0.01$ eV/Å, since the average change in energy between the two thresholds is only 0.1 kcal/mol, while the number of extra steps can be significant. We used $f_{\mathrm{max}} = 0.005$ eV/Å for eigenvector following performed on the five lowest-energy conformers. Note also that the algorithm is implemented in Cartesian coordinates. This makes it less efficient than the internal optimizer in xTB, which uses internal coordinates. Use of an internal coordinate optimizer may be of interest in the future.

Third, unlike in CREST[30], we did not constrain bond lengths with SHAKE[38]. Typically SHAKE is used to allow longer time steps and therefore accelerate the dynamics. Indeed, CREST uses a default time step of 5 fs, while the maximum value for unconstrained dynamics is typically 0.5 fs. However, the SHAKE implementation in ASE is rather slow, and therefore became a bottleneck instead of reducing run times. One can also increase the time step by artificially increasing the mass of hydrogen, since, as the lightest element, its motion is usually the fastest in the system. We followed the default in CREST and set the hydrogen mass to 4 amu. This allowed us to use a time step of 2 fs. Interestingly, we found that CREST automatically reduced the time step from 5 fs to 2 fs for TS conformer searches, since trial MTD runs with larger time steps all failed. This happened even though SHAKE is used in CREST. The same thing did not happen for conformer searches of equilibrium geometries. Hence our time step was the same as the CREST time step for TSs, even though we did not use SHAKE. Yet an efficient implementation of SHAKE is still of interest, since fixing the bond lengths during MTD might decrease the number of subsequent optimization steps needed.

Fourth, we fixed the CNNC atoms in each molecule when performing TS conformer searches. We experimented

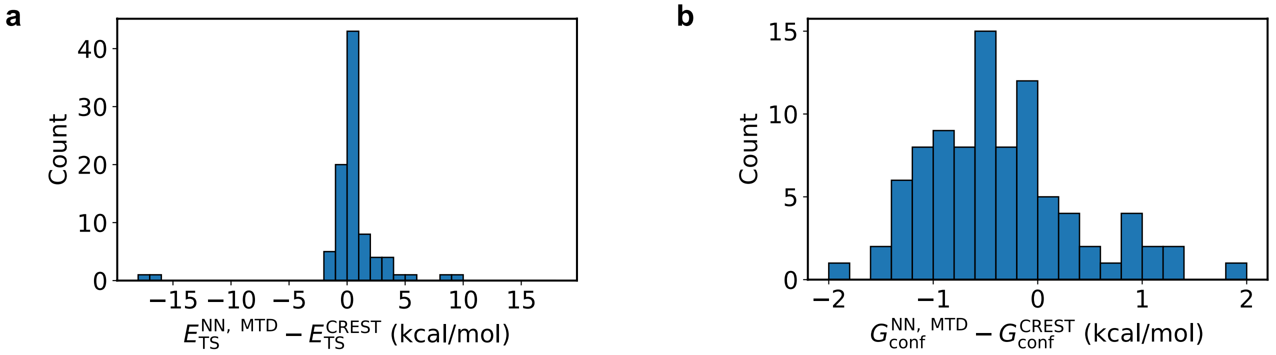

Figure S2: Energy differences between TSs generated with NN conformer searches and with CREST.

with Hookean constraints on the CNN angles and CNNC dihedrals, but this led to unstable dynamics. This was likely because the Cartesian gradient of an angle is undefined when the angle is 180°. We excluded the fixed atoms from the RMSD computation in MTD. For equilibrium geometries we excluded the CNNC atoms from the RMSD, but did not fix the atoms. Excluding these atoms ensured that *cis* did not isomerize to *trans*.

To evaluate our approach, we performed conformer searches with CREST and with our method on 100 relaxed scan TS geometries. We then optimized each CREST geometry with the NN, removed duplicates, and retained the geometries with energies under 2.5 kcal/mol. The top 5 conformers from each method were then optimized with eigenvector following. We checked that the mechanism of each TS matched the target mechanism of the relaxed scan (e.g. rotational TS for a rotation relaxed scan). We removed one species that had relaxed to an inversion TS with CREST after being seeded with a rotational TS. The distribution of TS energy differences is shown in Fig. S2(a). The mean value of $E_{\mathrm{TS}}^{\mathrm{NN,\ MTD}} - E_{\mathrm{TS}}^{\mathrm{CREST}}$ is 0.28 kcal/mol. The mean increases to 0.48 kcal/mol after removing outliers with absolute energy differences over 6 kcal/mol. The agreement between the two methods is quite good, despite using only one MTD simulation in the NN approach instead of 14. Further, the error in the $\Delta E^{\dagger}$ is expected to be smaller than the error in E_{TS}. This is because the conformer search can only overestimate the lowest energy, not underestimate it, and so some error cancellation can be expected.

We also compared the completeness of the ensembles in each of the two methods. To do so we computed the conformational free energy, defined in SI Sec. S3.6. Figure S2(b) shows the difference in conformational free energy between the two methods. Interestingly, we see that the NN generates a more complete ensemble on average. The mean value of $G_{\mathrm{conf}}^{\mathrm{NN,\ MTD}} - G_{\mathrm{conf}}^{\mathrm{CREST}}$ is -0.36 kcal/mol. This trend persists even when the lowest-energy conformers are quite close in energy between the two methods. For example, restricting ourselves to species in which the $|E_{\mathrm{TS}}^{\mathrm{NN,\ MTD}} - E_{\mathrm{TS}}^{\mathrm{CREST}}| \leq 0.5$ kcal/mol, we find that the difference in G_{conf} is -0.60 kcal/mol. We conclude that the NN ensembles are quite complete, and that the associated G_{conf} is reliable.

The NN approach is faster than CREST, mainly because fewer MTD runs are performed. NN energy and force calculations also tend to be faster than xTB for large molecules. A precise comparison is difficult, because xTB is run on CPUs and NNs are run on GPUs. This means that one cannot simply compare wall-clock times using equivalent hardware. Indeed, to compare the number of species that can be screened per day, one must also consider the number of GPUs available to the user vs. the number of CPUs. Nevertheless, some rough comparisons can still be made. For example, running 189 ps of MTD for a molecule with 102 atoms took 24 hours and 48 minutes with xTB on one Xeon-P8 core. If the xTB calculations were parallelized over all 48 cores on the node, then the run would take 31 minutes given perfect parallelization. Hence 1.9 MTD runs could be performed per node per hour. By contrast, the corresponding NN MTD run took 4 hours with one NVIDIA A100 GPU, using a batch of 50 species. This corresponds to 12.5 MTD runs per GPU per hour, or 25 per node per hour, assuming 2 GPUs per node. In this case, NN MTD is effectively $25/1.9 \approx 13$ times faster than xTB MTD.

Lastly, since the conformer search is the rate-limiting step in our workflow, any additional speed-ups would increase our throughput. While MTD enables exploration of conformational space, it is fundamentally limited by the fact that all steps must be taken in series. Uncorrelated geometries can only be generated after taking several hundred steps. Stochastic conformer generators are attractive because they can rapidly produce many uncorrelated structures. Examples include the ETKDG method in RDKit[39] and the commercial software Omega[40,41]. To adapt these generators for TSs, one would have to generate conformers of equilibrium geometries, and perform a relaxed scan from the equilibrium structures to the TS. Even if TS geometries would be generated directly, the structures would still have to be optimized. In either case, optimization would incur significant cost. Indeed, structure optimizations

Table S2: Parameters used in IRC searches. SD stands for steepest descent.

Parameter	Meaning	Value
`init_displ_de`	Target energy change for the first step	0.25 kcal/mol
`scale_displ_sd`	Factor for scaling the first SD step	0.15
`adapt_scale_disp`	Modify `scale_displ_sd` when the step size becomes smaller or larger	True
`sd_parabolic_fit`	Do a parabolic fit for finding the optimal SD step length	True
`interpolate_only`	Only allow interpolation for parabolic fit, not extrapolation	True
`do_sd_corr`	Apply a correction to the first SD step	True
`scale_displ_sd_corr`	Factor for scaling the correction to the SD step	0.33
`sd_corr_parabolic_fit`	Do a parabolic fit for finding the optimal SD step length	True
`tol_max_g`	Maximum gradient for convergence	2×10^{-3} Ha/Bohr
`tol_rms_g`	RMS gradient for convergence	5×10^{-4} Ha/Bohr

take the most time of any step in CREST and NN conformer generation. Therefore, avoiding MTD would not reduce the computation time to that of a stochastic conformer generator. Another approach is to train a generative model to produce TS geometries[42]. ML for equilibrium conformer generation has rapidly progressed in recent years[43–51], and many of the methods could also be applied to TS generation. These avenues would certainly be of interest in future work.

S3.3 Eigenvector following

EVF was implemented with Baker's rational function optimization method[52]. In the first step, the numerical Hessian was computed with finite differences, using the ASE vibrations package. We used a step size of 0.005 Å, and checked that the results were indistinguishable from the analytical Hessian computed with PyTorch. We used finite differences rather than automatic differentiation to minimize the consumed GPU memory. In subsequent steps the Hessian was updated using Powell's method[53]. We used a convergence threshold of $f_{\max} = 0.005$ eV/Å.

S3.4 Intrinsic reaction coordinate

We implemented an IRC algorithm based on the approach in Orca[54]. This approach is itself based on the method of Morokuma and coworkers[55]. Details can be found in the Orca 5.0.2 manual. The parameters that we used are given in Table S2. Note that the force tolerances are rather large, since only a loose optimization is needed to see if the IRC has the product and reactant on either end.

S3.5 Singlet-triplet crossing searches

Singlet-triplet crossings were located with a two-step procedure. In the first step, we performed IRC to locate the crossings one either side of the TS. In the second step, we optimized each of the two crossings by minimizing the energy while keeping the singlet-triplet gap close to zero. This yielded MECPs on each side of the TS.

In the first step we performed the normal IRC algorithm on the S_0 state. We also kept track of the T_1 energy, and stopped the optimization once $E_{T_1} - E_{S_0}$ changed sign. We then used the final geometry as the starting point for the MECP optimization.

The MECPs were optimized with the approach of Ref[56]. In a regular optimization, the objective function is the energy E, and so its negative gradient is $\mathbf{F}$. In an MECP optimization, the objective function is the energy subject to the constraint that the singlet-triplet gap is zero. We therefore used the following effective forces in the optimization:

$$\mathbf{F}' = \mathbf{P}\bar{\mathbf{F}} + \frac{1}{\alpha}\,\Delta E\,\Delta\mathbf{F}. \tag{S6}$$

The first term is the mean of the singlet and triplet forces, $\bar{\mathbf{F}}$, with the gradient of the energy gap ΔE projected out by $\mathbf{P}$. Its effect is to minimize the average singlet and triplet energies without increasing their gap. The second term contains the force difference $\Delta\mathbf{F}$. Since it is scaled by the energy difference ΔE, its effect is to minimize the

singlet-triplet gap. α is a constant with units of energy. The terms are given by

$$\Delta E = E_{T_1} - E_{S_0} \tag{S7}$$

$$\Delta \mathbf{F} = \mathbf{F}_{T_1} - \mathbf{F}_{S_0} \tag{S8}$$

$$\bar{\mathbf{F}} = \frac{1}{2}\left(\mathbf{F}_{T_1} + \mathbf{F}_{S_0}\right) \tag{S9}$$

$$\mathbf{P}\bar{\mathbf{F}} = \bar{\mathbf{F}} - \frac{\Delta \mathbf{F}^T \bar{\mathbf{F}}}{\Delta \mathbf{F}^T \Delta \mathbf{F}} \Delta \mathbf{F}, \tag{S10}$$

where a superscript T denotes transposition.

We implemented this approach in ASE, using a calculator that produced the forces of Eq. (S6). We then performed the optimization using the BFGS algorithm in ASE. The maximum force tolerance for convergence was set to 0.005 eV/Å. The constant α was set to 12.55 kcal/mol following a related MECP algorithm for conical intersections[57]. We found that the optimized MECPs were insensitive to the choice of α. For example, nearly identical MECP energies were obtained when using $\alpha = 1.0$ kcal/mol.

S3.6 Free energy calculations

The free energy was computed for reactants, products, and TSs, using

$$G = H - TS, \tag{S11}$$

where G is the Gibbs free energy, H is the enthalpy, and S is the entropy. The enthalpy was computed as

$$H = E_{\mathrm{el}} + E_{\mathrm{ZPE}} + E_{\mathrm{vib}}(T) + 4\, k_{\mathrm{B}}T + E_{\mathrm{conf}}, \tag{S12}$$

where the last term is the average conformer energy,

$$E_{\mathrm{conf}} = \sum_i p_i E_i, \tag{S13}$$

and the sum is over all conformers. p_i is the Boltzmann probability for conformer i, given by $p_i \propto \exp(-E_i/k_{\mathrm{B}}T)$. E_{el} is the electronic energy, E_{ZPE} is the zero-point energy, and $E_{\mathrm{vib}}(T)$ is the thermal vibrational energy. Standard expressions for E_{ZPE} and $E_{\mathrm{vib}}(T)$ can be found in Ref. [13]. Apart from E_{conf}, all terms in Eq. (S12) were computed using the ASE thermochemistry package.

The entropy was computed as

$$S = S_{\mathrm{mRRHO}} + S_{\mathrm{conf}}, \tag{S14}$$

where S_{mRRHO} is the modified rigid rotor-harmonic oscillator entropy (see below), and S_{conf} is the conformational entropy:

$$S_{\mathrm{conf}} = -k_{\mathrm{B}} \sum_i p_i \ln p_i. \tag{S15}$$

The sum is over all conformers of all four mechanisms when using TS theory, but only over rotational conformers when using the ISC approach. Note that if two mechanisms are the same by symmetry, then the conformational entropy is increased by $kT \log 2$, while ΔG is lowered by this amount. This is the same factor that would arise from using the symmetry number $\sigma = 2$ in the calculations[58].

The modified rigid rotor harmonic oscillator approximation[59] interpolates between a free rotor at low frequencies and a harmonic oscillator at high frequencies. This is more physically sound than a standard harmonic approximation, and avoids the divergent entropy of low-frequency harmonic modes. The rotor cutoff frequency was set to 50 cm^{-1}, following the default in xTB[34]. All other parameters were unchanged from Ref. [59].

Harmonic modes and frequencies were computed in the normal way[60] by diagonalizing the mass-weighted Hessian after projecting out rotation and translation. For reactants and products we checked that there were no imaginary frequencies. For TSs we checked that there was only one imaginary frequency, with magnitude ≥ 200 cm^{-1}. Species with reactants or products not satisfying these conditions were removed. Species were kept if they had at least one TS geometry from each mechanism satisfying these conditions.

For G^{X}, H^{X}, and S^{X} we used thermostatistical quantities from the rotational TSs. In principle the vibrational terms should be computed with the average Hessian of the singlet and triplet states at the crossing[56]. However, we found that this approach led to a sensitive dependence on α in Eq. (S6), and so we used the rotational TS results

instead.

S4 Non-adiabatic transition state theory

The reaction rate from non-adiabatic transition state theory (NA-TST) is given by[56]

$$k_{\mathrm{NA-TST}} = \frac{Z_{\mathrm{X}}}{hZ_{\mathrm{R}}} \int_0^{\infty} d\varepsilon\, P(\varepsilon)\, \exp(-\beta\varepsilon). \tag{S16}$$

Here $\beta = 1/(k_{\mathrm{B}}T)$, Z_{X} and Z_{R} are the rovibrational partition functions at the crossing point and reactant geometry, respectively, and $P(\varepsilon)$ is the probability of transitioning between the two electronic states at energy ε. $P(\varepsilon)$ can be computed within the Wentzel-Kramers-Brillouin (WKB) approximation[61,62]. The result is[63]

$$k_{\mathrm{NA-TST}} = k_{\mathrm{ISC}}\, \exp(-\beta\Delta G^{X}), \tag{S17}$$

where the intersystem crossing rate is

$$k_{\mathrm{ISC}} = \frac{\pi^{3/2}\alpha}{2h\sqrt{\lambda/(k_{\mathrm{B}}T)}} \left[1 + \frac{1}{2}\exp\left(\frac{1}{12\,\alpha^2\,(k_{\mathrm{B}}T\lambda)^3}\right)\right], \tag{S18}$$

and

$$\alpha = \frac{4H_{\mathrm{SO}}^{3/2}}{\hbar}\left(\frac{\mu}{F_g|\Delta\mathbf{F}|}\right)^{1/2} \tag{S19}$$

$$\lambda = \frac{|\Delta\mathbf{F}|}{2F_g H_{\mathrm{SO}}} \tag{S20}$$

$$F_g = \left|\sum_{j=1}^{N}\sum_{n=1}^{3} (\mathbf{F}_{S_0})_{jn}\, (\mathbf{F}_{T_1})_{jn}\right|^{1/2}. \tag{S21}$$

Here H_{SO} is the spin-orbit coupling, μ is the reduced mass of the reaction coordinate, F_g is the geometric mean of the singlet and triplet forces at the crossing, N is the number of atoms, and $\Delta\mathbf{F}$ is the force difference (Eq. (S8)). In NA-TST, the reaction coordinate is the direction of $\Delta\mathbf{F}$. The reduced mass is then given by

$$\mu = \left(\frac{1}{|\Delta\mathbf{F}|^2}\sum_{j=1}^{N}\sum_{n=1}^{3} \Delta\mathbf{F}_{jn}^2 m_n^{-1}\right)^{-1}, \tag{S22}$$

where m_n is the mass of the nth atom. Notice that Eq. (S18) scales quadratically with H_{SO}, as expected from Fermi's golden rule[64]. In re-expressing the equation from Ref.[63] we have used the relation $G_{\mathrm{rv}} = -k_{\mathrm{B}}T\log Z_{\mathrm{rv}}$ for the rovibrational free energy, and written $G^{\mathrm{X}} = E^{\mathrm{X}} + G_{\mathrm{rv}}^{\mathrm{X}}$. We have also corrected two typos (Eq. (7) in Ref.[63] should not contain $k_{\mathrm{B}}T$, and F_g involves a square root rather than a square).

In this work we have approximated H_{SO} as constant among all azobenzene derivatives. We tested this approximation by computing H_{SO} at the rotational TS of several derivatives. We included a derivative with chlorine, since, as a relatively heavy third row element, it could increase the coupling. Couplings are available in Q-Chem for TDFFT, but not for SF-TDDFT or CASPT2. They are also not available for CASPT2 in Orca. We therefore used TDDFT with both the B3LYP and BHHLYP functionals, and found that $H_{\mathrm{SO}} \approx 40$ cm^{-1}, with a relative range of 25% between the smallest and biggest values. The coupling is within a factor of two of the (14, 12) CASPT2 result[65], and the small variation indicates that $H_{\mathrm{SO}} \approx$ const. is a good approximation. It also suggests that the couplings from CASPT2 would be close to constant. In our calculations we therefore set $H_{\mathrm{SO}} = 20$ cm^{-1}, following the (presumably) more accurate CASPT2 result.

It is informative to estimate the impact of this constant coupling approximation. To do so, we note that the ISC rate scales quadratically with the spin-orbit coupling. Thus a relative change of 25% leads to a 56% change in the rate. Converting this to an effective activation entropy, and using $t_{\mathrm{ISC}} = 4.2$ ps as discussed below, gives a change of 0.26 kcal/mol to ΔG^{eff}. Thus the maximum error from this approximation is only 0.26 kcal/mol, and is thus negligible compared to other sources of error.

The ISC rate must be multiplied by two for the singlet $\to$ triplet transition, and by three for the triplet $\to$ singlet transition[65]. Using Eq. (S18) together with $H_{\mathrm{SO}} = 20$ cm^{-1}, we found that $t_{\mathrm{ISC}} = 5.7$ ps for $S \to T$ and 4.2 ps for $T \to S$. These are within a factor of two of the results quoted in Ref.[65] using Fermi's golden rule, thus

validating both their approach and ours. Note that we use the same spin-orbit coupling constant as Ref. [65], and so the differences come solely from the different rate formulas. The benefit of Eq. (S18) is that it does not contain the Franck-Condon factors used in Fermi's golden rule expressions [65,66]. Such terms are computationally expensive, and cannot be easily computed for anharmonic modes such as the reaction coordinate. We found that t_{ISC} was essentially constant among derivatives: the minimum time was 4.8 ps, and the maximum time was 5.8 ps.

S5 Connection between non-adiabatic and Eyring transition state theory

The intersystem crossing rate in NA-TST can be connected to the experimental $\Delta S^{\dagger}$. Experimentally one starts by assuming an Arrhenius transition rate,

$$k_{\mathrm{A}} = A e^{-E_a/(k_{\mathrm{B}}T)}. \tag{S23}$$

Here E_a is the activation energy and A is the Arrhenius prefactor. The two parameters are obtained experimentally from a plot of $\log k$ vs. $1/T$ [58]. Differentiating $\log k$ with respect to $1/T$ in Eqs. (2) and (S23), equating the two results, and invoking Eq. (2) yields [58]

$$\Delta S^{\dagger} = k_{\mathrm{B}} \left(\log \left(A \frac{h}{k_{\mathrm{B}}T} \right) - 1 \right), \tag{S24}$$

where the slight temperature dependence of S and H has been neglected. If the process is actually mediated by ISC, then we can apply the same logic to Eq. (S17), which yields

$$E_a = \Delta H^{\mathrm{X}} + \frac{k_{\mathrm{B}}T}{2} - \frac{3\eta k_{\mathrm{B}}}{T^2} \frac{\mathrm{e}^{\eta/T^3}}{2 + \mathrm{e}^{\eta/T^3}} \tag{S25}$$

$$\eta = \frac{1}{12\alpha^2 k_{\mathrm{B}}^3 \lambda^3}. \tag{S26}$$

Inserting Eqs. (S25) and (S23) into (S17) then gives

$$A_{\mathrm{NA-TST}} = k_{\mathrm{ISC}} \exp\left(\frac{\Delta S^{\mathrm{X}}}{k_{\mathrm{B}}} + \frac{1}{2} \right) \exp\left(\frac{-3\eta \, e^{\eta/T^3}}{(2 + e^{\eta/T^3})T^3} \right). \tag{S27}$$

Typically $\eta/T^3 \ll 1$, and so the last exponential in Eq. (S27) is of order one. Neglecting this term and inserting the result into Eq. (S24) gives the effective activation entropy,

$$\Delta S^{\mathrm{eff}} = k_{\mathrm{B}} \left(\log \frac{h k_{\mathrm{ISC}}}{k_{\mathrm{B}}T} - \frac{1}{2} \right) + \Delta S^{\mathrm{X}}. \tag{S28}$$

This is the entropy that would be inferred from experiment, given a triplet-mediated process with intersystem crossing rate k_{ISC}, and an entropy difference ΔS^{X} between the intersection geometry and the reactant geometry.

S6 Activation free energies by mechanism

SI Fig. S3 compares the activation free energies of the different mechanisms. The plots are for the 19,000 compounds screened in the main text. Panel (a) compares triplet-mediated isomerization with standard S_0 isomerization. ΔG^{eff} is lower than $\Delta G^{\dagger}$ in 65% of cases. This indicates that, at the SF-TDDFT level of theory, isomerization proceeds through T_1 for roughly 2/3 of the molecules. However, this result should be interpreted with caution. As shown in Table S3, SF-TDDFT overestimates the energy at both the MECP and the TS relative to the highly accurate SF-EOM-CCSD(dT). The overestimate is 5.4 kcal/mol at the MECP and 3.9 kcal/mol at the TS. This means that $\Delta G^{\mathrm{eff}} - \Delta G^{\dagger}$ is overestimated by 1.5 kcal/mol. We therefore expect that, for the 19,000 compounds screened, ΔG^{eff} should be lower than $\Delta G^{\dagger}$ in *more* than 65% of cases. Indeed, if we assume that these overestimates are constant among species, and thus subtract 1.5 kcal/mol from all ΔG^{eff}, we find that the triplet mechanism is preferred in 88% of cases. This result should also be interpreted with caution, however, since it neglects errors in ΔS^{eff} and $\Delta S^{\dagger}$, and assumes that the energy error is constant among all species.

Panel (b) compares the rotation and inversion mechanisms in TST. $\Delta G^{\dagger}_{\mathrm{rot}}$ is lower than $\Delta G^{\dagger}_{\mathrm{inv}}$ in 55% of all cases. Interestingly, this means that there is no strong preference for either mechanism at the SF-TDDFT level of

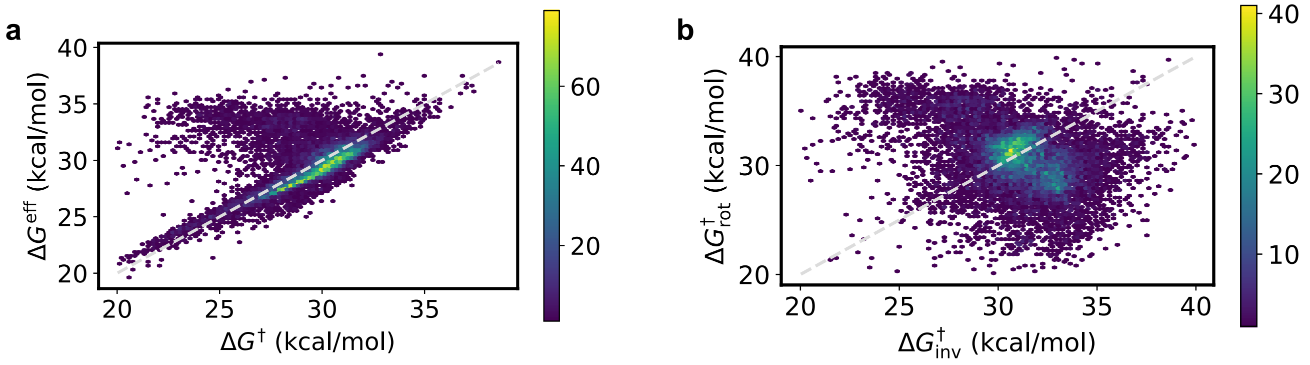

Figure S3: Reaction barriers for different mechanisms. (a) ΔG^{eff} from ISC vs. $\Delta G^{\dagger}$ from TST on the S_0 surface. (b) $\Delta G^{\dagger}$ from TST for rotation vs. inversion.

theory. This should be contrasted with the species in Fig. 4, for which rotation was preferred in every case.

S7 Thermal isomerization rates

SI Fig. S4 compares predicted and experimental thermal lifetimes, rather than ΔG values. It contains the same information as Fig. 4 in the main text, but with ΔG converted to a rate using Eq. (2), with $T = 298.15$ K. The lifetime is the reciprocal of the rate. Notice that we first converted experimental rates to ΔG, given the temperature of the experiment, and then back to a rate using $T = 298.15$ K. This ensures that the lifetimes are all compared at the same temperature. The line of best fit was computed for ΔG, and then converted to a lifetime.

We see that the experimental lifetimes span five orders of magnitude. Species **1** has the shortest lifetime at 10 minutes, while species **6** has the longest lifetime at 2.5 years. The systematic overestimation of the energies at the MECP and the TS leads to an overestimate of the lifetimes. The predicted lifetimes are respectively 4, 5, and 7 orders of magnitude too high for TST, ISC, and TST with only inversion. However, since the errors are systematic, the model still has a high Spearman rank correlation with experiment. Converting from ΔG to lifetime does not affect the ranking, and so the Spearman rank coefficients are the same as in Fig. 4. R^2 is negative because of the exponential dependence of lifetime errors on ΔG errors.

S8 Training

S8.1 Singlet models

Three different models were pre-trained on 680,736 gas-phase SF-TDDFT/6-31G* calculations from Ref. [3]. 95% of the data was used for training, 4% was used for validation, and 1% for testing. Each model was initialized with a different random seed and trained on different train/validation/test splits. Each model was then fine-tuned with SF-TDDFT/6-31G* using a C-PCM model of water [67–69]. The final models were trained on 42,938 geometries, with 500 used for validation and 500 for testing. Different splits were used to fine-tune each of the final models.

The singlet ground-state corresponds to an excitation from a triplet reference state in SF-TDDFT. We identified two singlets as the two states with the lowest $\langle S^2 \rangle$ of the three-lowest energy excitations [3]. The lower energy singlet was then taken as the ground singlet. Geometries with $\langle S^2 \rangle > 1$ in the ground singlet state were discarded. The spin contamination in the training set was fairly low, with mean$\langle S^2 \rangle = 0.16$.

Approximately 6% of species in the training set had non-zero charge (+1, +2, or +4). The model does not use charge in the input; however, all charge states in this work can be inferred from the number of bonds for each atom. For example, if nitrogen has a single bond to four different atoms, then it must have a partial charge of +1 to have a full octet. Since bonds can be inferred from atom type and distances [5], the model can learn the effect of different charge states from positions and atomic numbers alone.

Training was performed over energies and forces/force couplings in units of kcal/mol and kcal/mol/Å, respectively. Per-species reference energies were subtracted from each energy. These were obtained by summing atomic reference energies, computed using multi-variable linear regression from (atom type, count) to relaxed geometry energy computed with SF-TDDFT in vacuum. The set of vacuum reference energies was used for both vacuum and solvent training.

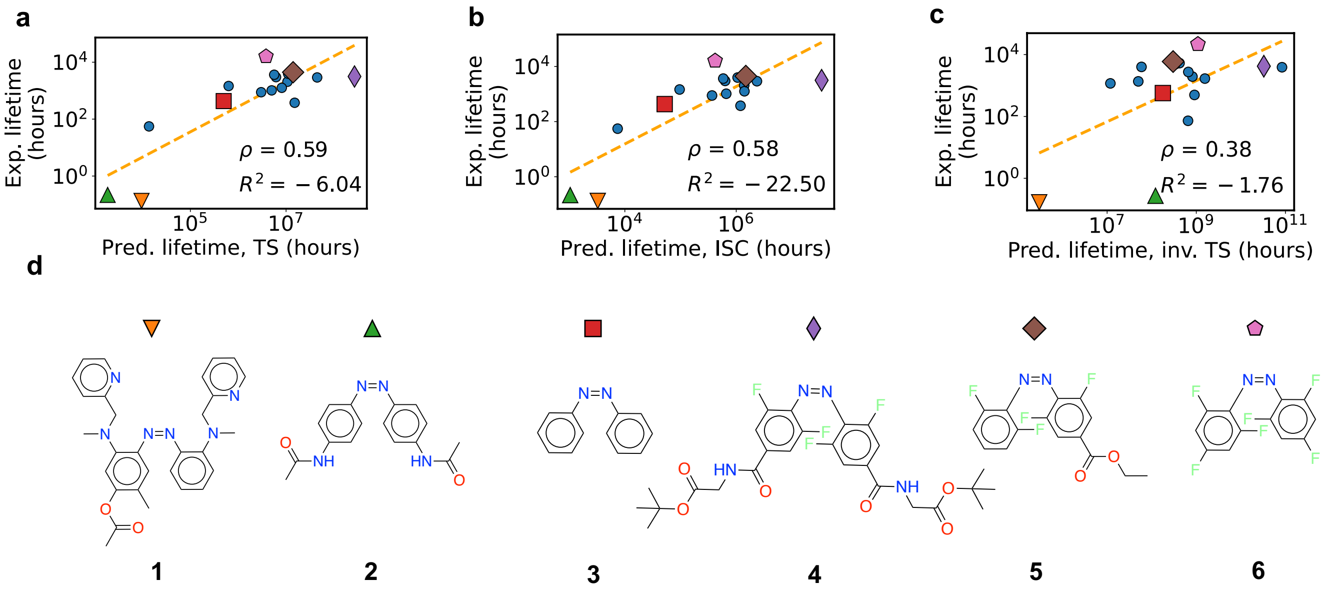

Figure S4: Experimental vs. predicted thermal lifetimes. Dotted orange lines are linear regression results from predicted to experimental ΔG, converted to a lifetime. ρ denotes the Spearman rank correlation. R^2 is computed between the regression results and the experimental data. (a) Prediction accuracy using TS theory. (b) Prediction accuracy using intersystem crossing. (c) As in (a), but with only the inversion mechanism. (d) Selected compounds highlighted in panels (a)-(c).

Configurations with 10-σ energy outliers were removed prior to training. Those with forces $\geq$450 kcal/mol/Å from the mean or energies $\geq$900 kcal/mol from the mean were also removed. Geometries were not removed on the basis of X-σ force deviations, since many geometries were close to TSs and hence had near-zero forces.

Models were trained with the Adam algorithm, using a batch size of 60 for the gas-phase models and 20 for the fine-tuned models. We used an MAE loss for the forces and energies:

$$\mathcal{L} = \frac{1}{M} \sum_{i=1}^{M} \rho_E \left| E_i - \hat{E}_i \right| + \frac{1}{3\sum_{i=1}^{M} N_i} \sum_{i=1}^{M} \sum_{j=1}^{N_i} \sum_{n=1}^{3} \rho_F \left| F_{j,n} - \hat{F}_{j,n} \right|. \tag{S29}$$

Here $\rho_E = 0.2$ is the energy weight, $\rho_F = 1.0$ is the force weight, M is the number of geometries in a batch, N_i is the number of atoms in geometry i, $\hat{X}$ is a predicted quantity and X is its true value. We used an MAE loss instead of a mean-squared-error loss, since this led to significantly better model performance.

The learning rate was initialized to 10^{-4} and reduced by a factor of two if the validation loss had not improved in X epochs. X was set to 10 for the gas-phase models and 50 for the fine-tuned models. Training was stopped when the learning rate fell below 10^{-5}. The final model was selected as the one with the lowest validation loss. Training was performed on a single 32 GB Nvidia Volta V100 GPU, and took approximately 2 days for each of the models.

S8.2 Triplet models

Triplet models were trained on the S_0/T_1 gap. They were pre-trained using energies from Ref.[3]. Gradients were not used since they had not been computed. For fine-tuning we computed gradients of both the singlet and triplet state for all geometries, and thus used both the gap and its gradient for training. The same loss tradeoff was used as for the singlet model. The learning rate was set 5×10^{-5} for pre-training and 10^{-4} for fine-tuning; the former was chosen because of instabilities in pre-training. To identify the triplet state, we first identified the two singlet states from the three lowest-energy excitations using the method above. The triplet state was then the remaining one. Data points with $\langle S^2 \rangle < 2.0$ or $\langle S^2 \rangle > 2.4$ were discarded.

SF-TDDFT uses an $M_S = 1$ triplet reference state. The excited $M_S = 0$ triplet and the reference $M_S = 1$ triplet should have the same energy. We have found this to be true for spin-flip coupled-cluster methods, but not for SF-TDDFT. This non-zero energy difference was also observed in Ref.[70]. The authors explained that the difference is due to orbital relaxation, since the orbitals are relaxed for the reference state but not for the TDDFT states.

It is not immediately clear which triplet energy should be used for ISC. We tested results using the reference triplet, the excited triplet, and an average of the two. We compared E^X to that of spin-flip coupled cluster for

azobenzene (SI Sec. S12), and found that the best agreement was for the triplet average. The excited triplet overestimated E^{X} by 5 kcal/mol, and the reference triplet underestimated it by nearly the same amount. However, we found that the correlation with experiment was slightly better when we used the SF-TDDFT triplet. Further, this is more consistent with the principle of treating singlet and triplet states on "equal footing". We therefore trained our models on the excited-state triplet energies. The same network architecture and training parameters were used as for the singlet models.

S8.3 Singlet excitation model

The singlet excitation model was trained directly on the S_0/S_1 gap. It was first pre-trained using energies and gradients from Ref.[3]. It was then fine-tuned using energies only, since we did not compute S_1 gradients on the new geometries.

S9 Filtering the screening results

We only kept the species that satisfied the following conditions:

1. **Endpoints are done**. Both *cis* and *trans* must have conformer ensembles and a minimum-energy geometry with no imaginary frequencies.
2. **Endpoints maintain *cis/trans* isomerism**. The optimized *cis* and *trans* isomers must actually be *cis* and *trans*, respectively. This is checked with RDKit.
3. **TS conformer generation is finished for each mechanism**. A TS conformer ensemble must be generated for each of the four mechanisms.
4. **At least one TS is converged for each mechanism**. To be converged, the TS must have only one imaginary frequency, and its magnitude must exceed 200 cm^{-1}.
5. **Graph is unchanged**. All of the TS and endpoint geometries must have the same molecular graph as the input SMILES. This is checked using D3 coordination numbers. Atom pairs with coordination numbers ≥ 0.95 are assigned a bond. The resulting bond list is checked against that of the original SMILES string.
6. **Vibrational frequencies are reliable**. A frequency calculation is deemed reliable if $|\Delta G^{\dagger} - \Delta E^{\dagger}| \leq 10$ kcal/mol.
7. **For the two singlet-triplet MECPs, one is closer to *cis* and the other to *trans***. This is checked using the root-mean-square displacement of the CNNC atoms after alignment.
8. **Each rotational TS connects to *cis* and *trans* through the IRC**. This is true if condition 7 is satisfied.

25,000 species were screened in total, and 18,877 remained after applying these filters.

S10 Experimental data

Most papers reported either the thermal half-life $\tau_{1/2}$, or the thermal lifetime τ. These are related through $\tau = (e/2)\,\tau_{1/2}$. In some cases the authors did not provide τ or $\tau_{1/2}$, but did plot the absorption spectrum at different time intervals. In these cases we inferred the thermal lifetime from the absorption plots.

In all cases we computed the activation free energy from Eyring TST using τ:

$$\Delta G^{\dagger} = k_{\mathrm{B}}T \log \frac{k_{\mathrm{B}}T\tau}{h}. \tag{S30}$$

Note that different works used different temperatures, and so T depends on the source.

S11 Note on mechanisms

S11.1 Concerted inversion

In our own calculations we have found that concerted inversion has two imaginary frequencies, and therefore is not a true TS. This is because it is a combination of two different inversions, each of which has one imaginary frequency. It is therefore higher in energy than a single inversion, not a true TS, and can be excluded from possible thermal mechanisms.

Table S3: Summary of the methods and results from our benchmark. $\Delta E^{\dagger}$ is the energy difference between the TS and the reactant, and ΔE^{X} is the energy difference between the singlet-triplet crossing and the reactant. They are shown schematically in Fig. 2 in the main text. DFT uses the B3LYP functional with D3BJ dispersion, while SF-TDDFT uses the BHHLYP functional, both with and without D3BJ dispersion. Energy differences are given in kcal/mol.

Method	Basis	Restricted?	Treatment of Static Correlation	Treatment of Dynamic Correlation	$\Delta E^{\dagger}$	ΔE^{X}
DFT	cc-pVDZ	Yes	Poor	Strong	35.6	n/a
DLPNO-UCCSD(T)	F12-cc-pV(D, T)Z extrap.	No	Poor	Very strong	42.1	n/a
SF-TDDFT	6-31G*	No	Moderate	Strong	31.5	27.8
SF-TDDFT (D3BJ)	6-31G*	No	Moderate	Strong	32.6	28.9
CASPT2 (14, 12)	cc-pVDZ	Yes	Strong	Moderate	25.4	18.7
SF-EOM-CCSD(dT)	6-31G*	Yes	Moderate	Very strong	28.7	23.5

S11.2 Inversion-assisted rotation

A relaxed scan from $\alpha \approx 120°$ to $\alpha \approx 180°$ will either end in a pure inversion TS or an inversion-assisted rotation TS, depending on the substituents. One can also be converted into the other through a conformational search with fixed CNNC atoms. Therefore, we grouped inversion and inversion-assisted rotation together.

S12 Quantum chemistry benchmark

Here we compare the torsional energy profiles of unsubstituted azobenzene predicted by different electronic structure methods. We analyze results from DFT, DLPNO-UCCSD(T) (domain based local pair-natural orbitals [71–80] for unrestricted coupled cluster with single, double, and perturbative triple excitations), SF-TDDFT, CASPT2 (complete active space with second-order perturbation theory), and SF-EOM-CCSD(dT) (spin-flip equation-of-motion coupled-cluster with single, double, and perturbative triple excitations [81]). The methods are summarized in Table S3. For DFT we use the B3LYP functional [82] with D3 [5] Becke-Johnson (BJ) dispersion [6]. For SF-TDDFT we use the BHHLYP functional both with and without D3BJ dispersion.

For B3LYP-D3BJ we use the double-zeta correlation-consistent (cc) basis set cc-pVDZ [83]. This model chemistry predicts activation free energies of azoarene isomerization that are in good agreement with experiment [84]. For DLPNO-UCCSD(T) we extrapolate to the complete basis-set limit using explicit correlation (F12) [85] with double- and triple-zeta basis sets (cc-pV(D, T)Z). Such high-accuracy basis sets can be used because of the computational efficiency of DLPNO. For BHHLYP SF-TDDFT we use the 6-31G* basis [86], since this is a medium-cost, medium-accuracy basis set that can be used to generate large amounts of data for ML. Similar quality basis-sets are used for CASPT2 and SF-EOM-CCSD(dT) due to computational constraints. B3LYP-D3BJ, CASPT2, and DLPNO-CCSD(T) calculations are performed with Orca 5.2 [54]. For DFT and CCSD(T) calculations in Orca we use the resolution of identity [87–93] and chain-of-spheres [94] approximations (`RIJCOSX`). The `TightPNO` setting is used for DLPNO calculations. All other calculations are performed with Q-Chem 5.3.

We performed a relaxed scan from $\omega = 0$ to $\omega = 180°$ in 1° steps using our trained NN potential. We constrained the dihedral angle at each step using a force constant of 1 Ha. We then performed single-point gas-phase calculations with the methods given above. Calculations were performed on all geometries, except for SF-EOM-CCSD(dT), which was performed on only nine geometries due to high computational cost. Those points are shown as stars, and are connected by a smooth line using a quadratic interpolation. Triplet calculations with DLPNO-CCSD(T) failed to converge and are hence not reported.

Results are shown in Fig. S5. The plots contain several salient features. First, the single-reference methods DFT and UCCSD(T) predict a cusp near $\omega = 90°$, while the multi-reference methods produce a smooth maximum of lower energy. This can be seen in Fig. S5(d), where DFT (red) closely tracks UCCSD(T) (gray) until $\omega = 90°$. Both methods have a cusp at $\omega = 90°$, but the energy from DFT is lower. Similar results have been found for ethylene torsion [95].

As shown in Table S3, DFT gives an activation energy of 35.6 kcal/mol, while UCCSD(T) gives 42.1 kcal/mol. The cusp in the DFT energy explains why previous work could not optimize the rotational TS with DFT [58]. Optimization methods such as EVF [52] use a quadratic expansion of the PES to locate critical points. This expansion fails in the vicinity of a cusp, since the Hessian is undefined at this point.

Previous calculations with DFT found that inversion is the preferred mechanism in gas phase [13]. However, given that the DFT activation energy is 10 kcal/mol higher than CASPT2, such conclusions are not to be trusted. Indeed, the inversion activation energy was found to be 31 kcal/mol with CASPT2 (10, 8) [96], which is 6 kcal/mol higher

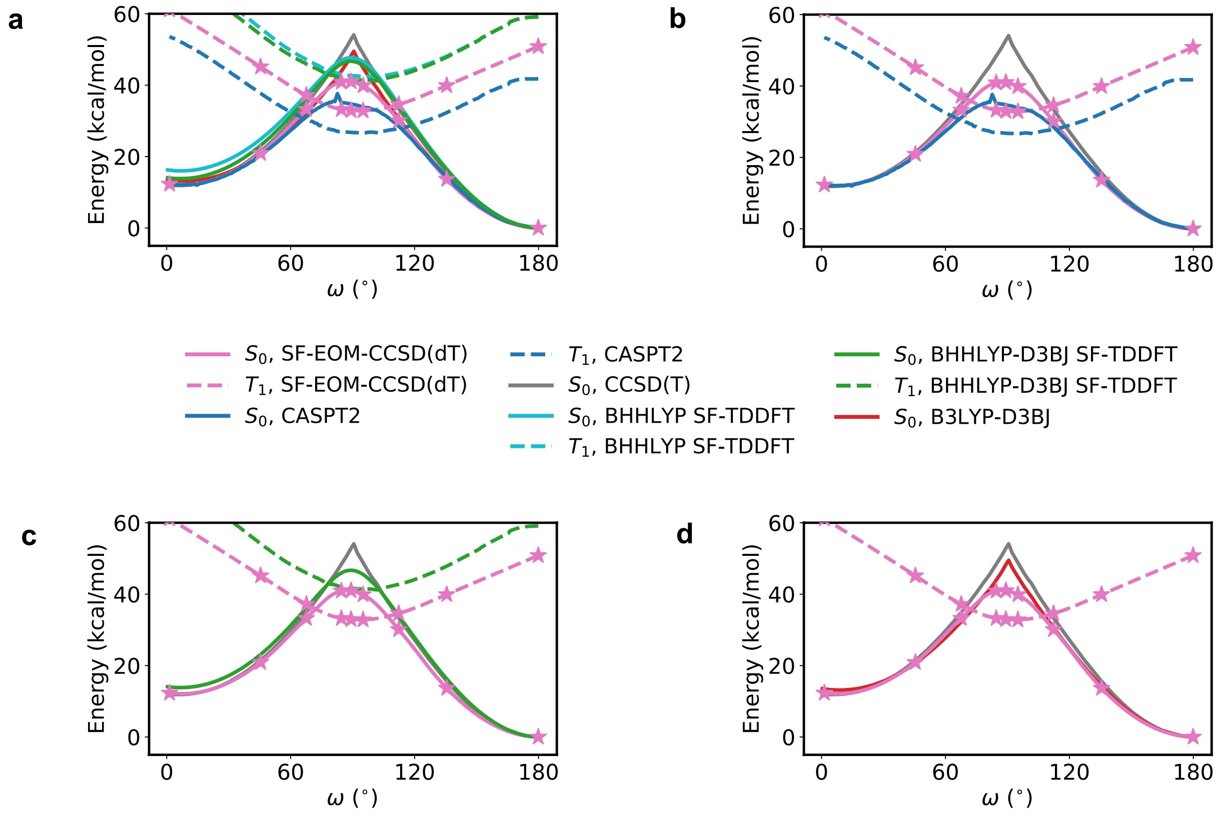

Figure S5: Torsional energy profile of azobenzene computed with different methods. Singlet and triplet energies are shown with full and dashed lines, respectively. Singlet and triplet energies computed with the same method are shown with the same color. (a) Results from all methods. For ease of visualization, different subsets of methods are compared in panels (b)-(d). (b) The multi-reference methods SF-EOM-CCSD(dT) and CASPT2 are compared with the single-reference method CCSD(T). (c) The multi-reference methods SF-EOM-CCSD(dT) and SF-TDDFT are compared with the single-reference method CCSD(T). (d) The multi-reference method SF-EOM-CCSD(dT) is compared with the single-reference methods B3LYP and CCSD(T).

than the CASPT2 rotation barrier here. Thus according to CASPT2, isomerization would proceed by rotation even if it were not mediated by ISC.

It is also interesting that the maximum value of the CCSD(T) T_1-diagnostic[97] is 0.013, which occurs at $\omega = 90°$. The minimum value is 0.01. A value above 0.02 indicates that the problem has multi-reference character, and that CCSD(T) should not be trusted. The diagnostic stays well below 0.02 despite the clear multi-reference nature of the problem. We also note that the unrestricted CCSD(T) results collapsed to the restricted results, with the square spin $\langle S^2 \rangle = 0$ for all geometries.

A second important result is that all multi-reference methods predict smooth TS maxima with singlet-triplet crossings on either side. Examples are CASPT2 and SF-EOM-CCSD(dT) in Fig. S5(b), and SF-EOM-CCSD(dT) and SF-TDDFT in Fig. S5(c). However, the $\Delta E^{\dagger}$ and ΔE^{X} predicted by the different methods are in quantitative disagreement. The activation energies predicted by CASPT2, SF-EOM-CCSD(dT), and SF-TDDFT (D3BJ) are 25.4, 28.7, and 32.6 kcal/mol, respectively. The singlet-triplet crossing energies are 18.7, 23.5, and 28.9 kcal/mol respectively. Given the disagreement between CASPT2 and SF-EOM, it is not immediately clear which method should be taken as the "ground truth".

On balance, however, it seems that SF-EOM-CCSD(dT) is likely more accurate. One reason is that its predictions are in quantitative agreement with MR-CISD+Q (multi-reference configuration interaction with singles and doubles, plus a Davidson correction) for a standard rotational conical intersection benchmark[98]. Its treatment of static correlation should therefore be sufficient for azobenzene, while its treatment of dynamic correlation is better than that of CASPT2. Moreover, its predicted activation enthalpy is in better agreement with experiment. The experimental activation enthalpy is 21.1 kcal/mol[99], which corresponds to ΔH^{X} in the ISC picture. Our NN calculations indicate that $\Delta H^{\mathrm{X}} \approx \Delta E^{\mathrm{X}} - 2.5$ kcal/mol for azobenzene. Therefore, $\Delta H^{\mathrm{X}} \approx 21.0$ kcal/mol with SF-EOM-CCSD(dT), while $\Delta H^{\mathrm{X}} \approx 16.2$ kcal/mol with CASPT2. The former agrees better with experiment than the latter.

A third important result is that SF-TDDFT's predictions are in qualitative agreement with other multi-reference methods, but not quantitative agreement. As in the other methods, SF-TDDFT predicts a smooth maximum with singlet-triplet crossings on either side of the TS. However, its predicted barrier is 3.9 kcal/mol higher than that of SF-EOM, and 7.2 kcal/mol higher than that of CASPT2. Its singlet-triplet crossing energy is 5.4 kcal/mol higher than SF-EOM, and 10.2 kcal/mol higher than CASPT2. These errors make it difficult to compare the ISC reaction rate with the Eyring reaction rate using SF-TDDFT. Hence in the main text we simply assume that all azobenzene derivatives isomerize through ISC.

Lastly, the plots show the importance of adding dispersion corrections to DFT. Figure S5(a) includes results of SF-TDDFT with and without dispersion. These are shown in green and cyan, respectively. When dispersion is included, all single- and multi-reference methods are in good agreement away from $\omega = 90°$. When dispersion is not included, SF-TDDFT overestimates the *cis* energy, because the attractive force between the two benzene rings is underestimated. Similar conclusions have been made in previous work[58,84]. There is still some residual error in the *cis* energy, even with dispersion, which does not occur with B3LYP-D3BJ. This is likely because the D3 parameters for BHHLYP were chosen for ground-state DFT, not SF-TDDFT.

References

(1) Schütt, K.; Unke, O.; Gastegger, M. Equivariant message passing for the prediction of tensorial properties and molecular spectra. International Conference on Machine Learning. 2021; pp 9377–9388.

(2) Paszke, A. et al. *Advances in Neural Information Processing Systems 32*; 2019; pp 8024–8035.

(3) Axelrod, S.; Shakhnovich, E.; Gómez-Bombarelli, R. Excited state non-adiabatic dynamics of large photoswitchable molecules using a chemically transferable machine learning potential. *Nature Communications* **2022**, *13*, 3440.

(4) Frey, N.; Soklaski, R.; Axelrod, S.; Samsi, S.; Gomez-Bombarelli, R.; Coley, C.; Gadepally, V. Neural Scaling of Deep Chemical Models. **2022**,

(5) Grimme, S.; Antony, J.; Ehrlich, S.; Krieg, H. A consistent and accurate ab initio parametrization of density functional dispersion correction (DFT-D) for the 94 elements H-Pu. *The Journal of chemical physics* **2010**, *132*, 154104.

(6) Grimme, S.; Ehrlich, S.; Goerigk, L. Effect of the damping function in dispersion corrected density functional theory. *Journal of computational chemistry* **2011**, *32*, 1456–1465.

(7) Paszke, A.; Gross, S.; Chintala, S.; Chanan, G.; Yang, E.; DeVito, Z.; Lin, Z.; Desmaison, A.; Antiga, L.; Lerer, A. Automatic differentiation in pytorch. **2017**,

(8) Unke, O. T.; Meuwly, M. PhysNet: A neural network for predicting energies, forces, dipole moments, and partial charges. *Journal of chemical theory and computation* **2019**, *15*, 3678–3693.

(9) PhysNet. `https://github.com/MMunibas/PhysNet`.

(10) Malkin, S.; Fischer, E. Temperature dependence of photoisomerization. Part II. Quantum yields of cis-trans isomerizations in azo-compounds. *The Journal of Physical Chemistry* **1962**, *66*, 2482–2486.

(11) Nagamani, S. A.; Norikane, Y.; Tamaoki, N. Photoinduced hinge-like molecular motion: studies on xanthene-based cyclic azobenzene dimers. *The Journal of organic chemistry* **2005**, *70*, 9304–9313.

(12) Sierocki, P.; Maas, H.; Dragut, P.; Richardt, G.; Vögtle, F.; De Cola, L.; Brouwer, F.; Zink, J. I. Photoisomerization of azobenzene derivatives in nanostructured silica. *The Journal of Physical Chemistry B* **2006**, *110*, 24390–24398.

(13) Dokic, J.; Gothe, M.; Wirth, J.; Peters, M. V.; Schwarz, J.; Hecht, S.; Saalfrank, P. Quantum chemical investigation of thermal cis-to-trans isomerization of azobenzene derivatives: substituent effects, solvent effects, and comparison to experimental data. *The Journal of Physical Chemistry A* **2009**, *113*, 6763–6773.

(14) Bandara, H. D.; Friss, T. R.; Enriquez, M. M.; Isley, W.; Incarvito, C.; Frank, H. A.; Gascon, J.; Burdette, S. C. Proof for the concerted inversion mechanism in the trans→cis isomerization of azobenzene using hydrogen bonding to induce isomer locking. *The Journal of organic chemistry* **2010**, *75*, 4817–4827.

(15) Bandara, H. D.; Cawley, S.; Gascón, J. A.; Burdette, S. C. Short-circuiting azobenzene photoisomerization with electron-donating substituents and reactivating the photochemistry with chemical modification. *Chemistry – A European Journal* **2011**, *2011*, 2916–2919.

(16) Beharry, A. A.; Sadovski, O.; Woolley, G. A. Azobenzene photoswitching without ultraviolet light. *Journal of the American Chemical Society* **2011**, *133*, 19684–19687.

(17) Knie, C.; Utecht, M.; Zhao, F.; Kulla, H.; Kovalenko, S.; Brouwer, A. M.; Saalfrank, P.; Hecht, S.; Bléger, D. *ortho*-Fluoroazobenzenes: Visible light switches with very long-lived Z isomers. *Chemistry–A European Journal* **2014**, *20*, 16492–16501.

(18) Schweighauser, L.; Strauss, M. A.; Bellotto, S.; Wegner, H. A. Attraction or repulsion? London dispersion forces control azobenzene switches. *Angewandte Chemie International Edition* **2015**, *54*, 13436–13439.

(19) Moreno, J.; Gerecke, M.; Grubert, L.; Kovalenko, S. A.; Hecht, S. Sensitized two-NIR-photon Z→E isomerization of a visible-light-addressable bistable azobenzene derivative. *Angewandte Chemie International Edition* **2016**, *55*, 1544–1547.

(20) Gutzeit, V. A.; Acosta-Ruiz, A.; Munguba, H.; Häfner, S.; Landra-Willm, A.; Mathes, B.; Mony, J.; Yarotski, D.; Börjesson, K.; Liston, C., et al. A fine-tuned azobenzene for enhanced photopharmacology in vivo. *Cell Chemical Biology* **2021**, *28*, 1648–1663.

(21) Lv, S.; Li, X.; Yang, L.; Ren, H.; Jiang, J. Computational design of photoswitchable anion receptors: Red-shifted and bistable di-ortho-fluoro di-ortho-chloro azobenzene derivatives. *Chemical Physics* **2021**, *548*, 111246.

(22) Konrad, D. B.; Savasci, G.; Allmendinger, L.; Trauner, D.; Ochsenfeld, C.; Ali, A. M. Computational design and synthesis of a deeply red-shifted and bistable azobenzene. *Journal of the American Chemical Society* **2020**, *142*, 6538–6547.

(23) RDKit: Open-source cheminformatics. `http://www.rdkit.org`.

(24) Larsen, A. H. et al. The atomic simulation environment—a Python library for working with atoms. *Journal of Physics: Condensed Matter* **2017**, *29*, 273002.

(25) Yue, L.; Liu, Y.; Zhu, C. Performance of TDDFT with and without spin-flip in trajectory surface hopping dynamics: cis⇌trans azobenzene photoisomerization. *Physical Chemistry Chemical Physics* **2018**, *20*, 24123–24139.

(26) Broyden, C. G. The convergence of a class of double-rank minimization algorithms 1. general considerations. *IMA Journal of Applied Mathematics* **1970**, *6*, 76–90.

(27) Fletcher, R. A new approach to variable metric algorithms. *The computer journal* **1970**, *13*, 317–322.

(28) Goldfarb, D. A family of variable-metric methods derived by variational means. *Mathematics of computation* **1970**, *24*, 23–26.

(29) Shanno, D. F. Conditioning of quasi-Newton methods for function minimization. *Mathematics of computation* **1970**, *24*, 647–656.

(30) Grimme, S. Exploration of chemical compound, conformer, and reaction space with meta-dynamics simulations based on tight-binding quantum chemical calculations. *Journal of chemical theory and computation* **2019**, *15*, 2847–2862.

(31) Pracht, P.; Bohle, F.; Grimme, S. Automated exploration of the low-energy chemical space with fast quantum chemical methods. *Physical Chemistry Chemical Physics* **2020**, *22*, 7169–7192.

(32) Grimme, S.; Bohle, F.; Hansen, A.; Pracht, P.; Spicher, S.; Stahn, M. Efficient quantum chemical calculation of structure ensembles and free energies for nonrigid molecules. *The Journal of Physical Chemistry A* **2021**, *125*, 4039–4054.

(33) Grimme, S.; Bannwarth, C.; Dohm, S.; Hansen, A.; Pisarek, J.; Pracht, P.; Seibert, J.; Neese, F. Fully automated quantum-chemistry-based computation of spin–spin-coupled nuclear magnetic resonance spectra. *Angewandte Chemie International Edition* **2017**, *56*, 14763–14769.

(34) Bannwarth, C.; Ehlert, S.; Grimme, S. GFN2-xTB–An accurate and broadly parametrized self-consistent tight-binding quantum chemical method with multipole electrostatics and density-dependent dispersion contributions. *Journal of chemical theory and computation* **2019**, *15*, 1652–1671.

(35) Spicher, S.; Grimme, S. Robust atomistic modeling of materials, organometallic, and biochemical systems. *Angewandte Chemie International Edition* **2020**, *59*, 15665–15673.

(36) Conformer-Rotamer Ensemble Sampling Tool. `https://github.com/grimme-lab/crest`.

(37) Liu, D. C.; Nocedal, J. On the limited memory BFGS method for large scale optimization. *Mathematical programming* **1989**, *45*, 503–528.

(38) Ryckaert, J.-P.; Ciccotti, G.; Berendsen, H. J. Numerical integration of the cartesian equations of motion of a system with constraints: molecular dynamics of n-alkanes. *Journal of computational physics* **1977**, *23*, 327–341.

(39) Riniker, S.; Landrum, G. A. Better informed distance geometry: using what we know to improve conformation generation. *Journal of chemical information and modeling* **2015**, *55*, 2562–2574.

(40) Hawkins, P. C.; Skillman, A. G.; Warren, G. L.; Ellingson, B. A.; Stahl, M. T. Conformer generation with OMEGA: algorithm and validation using high quality structures from the Protein Databank and Cambridge Structural Database. *Journal of chemical information and modeling* **2010**, *50*, 572–584.

(41) Hawkins, P. C.; Nicholls, A. Conformer generation with OMEGA: learning from the data set and the analysis of failures. *Journal of chemical information and modeling* **2012**, *52*, 2919–2936.

(42) Pattanaik, L.; Ingraham, J. B.; Grambow, C. A.; Green, W. H. Generating transition states of isomerization reactions with deep learning. *Physical Chemistry Chemical Physics* **2020**, *22*, 23618–23626.

(43) Xu, M.; Luo, S.; Bengio, Y.; Peng, J.; Tang, J. Learning neural generative dynamics for molecular conformation generation. International Conference on Learning Representations. 2020.

(44) Shi, C.; Luo, S.; Xu, M.; Tang, J. Learning gradient fields for molecular conformation generation. International Conference on Machine Learning. 2021; pp 9558–9568.

(45) Ganea, O.; Pattanaik, L.; Coley, C.; Barzilay, R.; Jensen, K.; Green, W.; Jaakkola, T. Geomol: Torsional geometric generation of molecular 3D conformer ensembles. *Advances in Neural Information Processing Systems* **2021**, *34*.

(46) Xu, M.; Wang, W.; Luo, S.; Shi, C.; Bengio, Y.; Gomez-Bombarelli, R.; Tang, J. An end-to-end framework for molecular conformation generation via bilevel programming. International Conference on Machine Learning. 2021; pp 11537–11547.

(47) Roney, J. P.; Maragakis, P.; Skopp, P.; Shaw, D. E. Generating realistic 3D molecules with an equivariant conditional likelihood model. **2021**,

(48) Luo, S.; Shi, C.; Xu, M.; Tang, J. Predicting molecular conformation via dynamic graph score matching. *Advances in Neural Information Processing Systems* **2021**, *34*.

(49) Jing, B.; Corso, G.; Barzilay, R.; Jaakkola, T. S. Torsional diffusion for molecular conformer generation. ICLR2022 Machine Learning for Drug Discovery. 2022.

(50) Xu, M.; Yu, L.; Song, Y.; Shi, C.; Ermon, S.; Tang, J. GeoDiff: A Geometric diffusion model for molecular conformation generation. International Conference on Learning Representations. 2021.

(51) Hoogeboom, E.; Satorras, V. G.; Vignac, C.; Welling, M. Equivariant diffusion for molecule generation in 3D. *arXiv preprint arXiv:2203.17003* **2022**,

(52) Baker, J. An algorithm for the location of transition states. *Journal of Computational Chemistry* **1986**, *7*, 385–395.

(53) Powell, M. J. Recent advances in unconstrained optimization. *Mathematical Programming* **1971**, *1*, 26–57.

(54) Neese, F.; Wennmohs, F.; Becker, U.; Riplinger, C. The ORCA quantum chemistry program package. *The Journal of Chemical Physics* **2020**, *152*, 224108.

(55) Ishida, K.; Morokuma, K.; Komornicki, A. The intrinsic reaction coordinate. An *ab initio* calculation for HNC $\rightarrow$ HCN and $H^- + CH_4 \rightarrow {CH_4}^+H^-$. *The Journal of Chemical Physics* **1977**, *66*, 2153–2156.

(56) Lykhin, A. O.; Kaliakin, D. S.; dePolo, G. E.; Kuzubov, A. A.; Varganov, S. A. Nonadiabatic transition state theory: Application to intersystem crossings in the active sites of metal-sulfur proteins. *International Journal of Quantum Chemistry* **2016**, *116*, 750–761.

(57) Levine, B. G.; Coe, J. D.; Martínez, T. J. Optimizing conical intersections without derivative coupling vectors: application to multistate multireference second-order perturbation theory (MS-CASPT2). *The Journal of Physical Chemistry B* **2008**, *112*, 405–413.

(58) Rietze, C.; Titov, E.; Lindner, S.; Saalfrank, P. Thermal isomerization of azobenzenes: on the performance of Eyring transition state theory. *Journal of Physics: Condensed Matter* **2017**, *29*, 314002.

(59) Grimme, S. Supramolecular binding thermodynamics by dispersion-corrected density functional theory. *Chemistry–A European Journal* **2012**, *18*, 9955–9964.

(60) Ochterski, J. W. Vibrational analysis in Gaussian. 1999; `https://gaussian.com/vib/`.

(61) Nikitin, E. E.; Kearsley, M. J. *Theory of Elementary Atomic and Molecular Processes in Gases*; Clarendon Press: Oxford, 1974.

(62) Delos, J. B. On the reactions of N_2 with O. *The Journal of Chemical Physics* **1973**, *59*, 2365–2369.

(63) Liu, S.; Srinivasan, S.; Tao, J.; Grady, M. C.; Soroush, M.; Rappe, A. M. Modeling spin-forbidden monomer self-initiation reactions in spontaneous free-radical polymerization of acrylates and methacrylates. *The Journal of Physical Chemistry A* **2014**, *118*, 9310–9318.

(64) Fermi, E. *Notes on quantum mechanics*; University of Chicago Press, 1995.

(65) Cembran, A.; Bernardi, F.; Garavelli, M.; Gagliardi, L.; Orlandi, G. On the mechanism of the cis-trans isomerization in the lowest electronic states of azobenzene: S_0, S_1, and T_1. *Journal of the American Chemical Society* **2004**, *126*, 3234–3243.

(66) Valiev, R.; Cherepanov, V.; Baryshnikov, G. V.; Sundholm, D. First-principles method for calculating the rate constants of internal-conversion and intersystem-crossing transitions. *Physical Chemistry Chemical Physics* **2018**, *20*, 6121–6133.

(67) Truong, T. N.; Stefanovich, E. V. A new method for incorporating solvent effect into the classical, ab initio molecular orbital and density functional theory frameworks for arbitrary shape cavity. *Chemical Physics Letters* **1995**, *240*, 253–260.

(68) Barone, V.; Cossi, M. Quantum calculation of molecular energies and energy gradients in solution by a conductor solvent model. *The Journal of Physical Chemistry A* **1998**, *102*, 1995–2001.

(69) Cossi, M.; Rega, N.; Scalmani, G.; Barone, V. Energies, structures, and electronic properties of molecules in solution with the C-PCM solvation model. *Journal of computational chemistry* **2003**, *24*, 669–681.

(70) Huix-Rotllant, M.; Natarajan, B.; Ipatov, A.; Wawire, C. M.; Deutsch, T.; Casida, M. E. Assessment of noncollinear spin-flip Tamm–Dancoff approximation time-dependent density-functional theory for the photochemical ring-opening of oxirane. *Physical Chemistry Chemical Physics* **2010**, *12*, 12811–12825.

(71) Neese, F.; Hansen, A.; Liakos, D. G. Efficient and accurate approximations to the local coupled cluster singles doubles method using a truncated pair natural orbital basis. *The Journal of chemical physics* **2009**, *131*, 064103.

(72) Neese, F.; Hansen, A.; Wennmohs, F.; Grimme, S. Accurate theoretical chemistry with coupled pair models. *Accounts of chemical research* **2009**, *42*, 641–648.

(73) Neese, F.; Wennmohs, F.; Hansen, A. Efficient and accurate local approximations to coupled-electron pair approaches: An attempt to revive the pair natural orbital method. *The Journal of chemical physics* **2009**, *130*, 114108.

(74) Liakos, D. G.; Hansen, A.; Neese, F. Weak molecular interactions studied with parallel implementations of the local pair natural orbital coupled pair and coupled cluster methods. *Journal of Chemical Theory and Computation* **2011**, *7*, 76–87.

(75) Hansen, A.; Liakos, D. G.; Neese, F. Efficient and accurate local single reference correlation methods for high-spin open-shell molecules using pair natural orbitals. *The Journal of chemical physics* **2011**, *135*, 214102.

(76) Riplinger, C.; Neese, F. An efficient and near linear scaling pair natural orbital based local coupled cluster method. *The Journal of chemical physics* **2013**, *138*, 034106.

(77) Riplinger, C.; Sandhoefer, B.; Hansen, A.; Neese, F. Natural triple excitations in local coupled cluster calculations with pair natural orbitals. *The Journal of chemical physics* **2013**, *139*, 134101.

(78) Riplinger, C.; Pinski, P.; Becker, U.; Valeev, E. F.; Neese, F. Sparse maps–A systematic infrastructure for reduced-scaling electronic structure methods. II. Linear scaling domain based pair natural orbital coupled cluster theory. *The Journal of chemical physics* **2016**, *144*, 024109.

(79) Datta, D.; Kossmann, S.; Neese, F. Analytic energy derivatives for the calculation of the first-order molecular properties using the domain-based local pair-natural orbital coupled-cluster theory. *The Journal of Chemical Physics* **2016**, *145*, 114101.

(80) Saitow, M.; Becker, U.; Riplinger, C.; Valeev, E. F.; Neese, F. A new near-linear scaling, efficient and accurate, open-shell domain-based local pair natural orbital coupled cluster singles and doubles theory. *The Journal of chemical physics* **2017**, *146*, 164105.

(81) Manohar, P. U.; Krylov, A. I. A noniterative perturbative triples correction for the spin-flipping and spin-conserving equation-of-motion coupled-cluster methods with single and double substitutions. *The Journal of chemical physics* **2008**, *129*, 194105.

(82) Becke, A. D. Density-functional thermochemistry. III. The role of exact exchange. *The Journal of chemical physics* **1993**, *98*, 5648.

(83) Dunning Jr, T. H. Gaussian basis sets for use in correlated molecular calculations. I. The atoms boron through neon and hydrogen. *The Journal of chemical physics* **1989**, *90*, 1007–1023.

(84) Adrion, D. M.; Kaliakin, D. S.; Neal, P.; Lopez, S. A. Benchmarking of density functionals for Z-azoarene half-lives via automated transition state search. *The Journal of Physical Chemistry A* **2021**, *125*, 6474–6485.

(85) Pavošević, F.; Peng, C.; Pinski, P.; Riplinger, C.; Neese, F.; Valeev, E. F. SparseMaps–A systematic infrastructure for reduced scaling electronic structure methods. V. Linear scaling explicitly correlated coupled-cluster method with pair natural orbitals. *The Journal of chemical physics* **2017**, *146*, 174108.

(86) Hehre, W. J.; Radom, L.; Schleyer, P. v. R.; Pople, J. *Ab initio* Molecular Orbital Theory. 1986.

(87) Whitten, J. L. Coulombic potential energy integrals and approximations. *The Journal of Chemical Physics* **1973**, *58*, 4496–4501.

(88) Baerends, E.; Ellis, D.; Ros, P. Self-consistent molecular Hartree-Fock-Slater calculations I. The computational procedure. *Chemical Physics* **1973**, *2*, 41–51.

(89) Dunlap, B. I.; Connolly, J.; Sabin, J. On some approximations in applications of Xα theory. *The Journal of Chemical Physics* **1979**, *71*, 3396–3402.

(90) Van Alsenoy, C. Ab initio calculations on large molecules: The multiplicative integral approximation. *Journal of computational chemistry* **1988**, *9*, 620–626.

(91) Kendall, R. A.; Früchtl, H. A. The impact of the resolution of the identity approximate integral method on modern ab initio algorithm development. *Theoretical Chemistry Accounts* **1997**, *97*, 158–163.

(92) Eichkorn, K.; Treutler, O.; Öhm, H.; Häser, M.; Ahlrichs, R. Auxiliary basis sets to approximate Coulomb potentials. *Chemical physics letters* **1995**, *240*, 283–290.

(93) Eichkorn, K.; Weigend, F.; Treutler, O.; Ahlrichs, R. Auxiliary basis sets for main row atoms and transition metals and their use to approximate Coulomb potentials. *Theoretical Chemistry Accounts* **1997**, *97*, 119–124.

(94) Neese, F.; Wennmohs, F.; Hansen, A.; Becker, U. Efficient, approximate and parallel Hartree-Fock and hybrid DFT calculations. A 'chain-of-spheres' algorithm for the Hartree–Fock exchange. *Chemical Physics* **2009**, *356*, 98–109.

(95) Shao, Y.; Head-Gordon, M.; Krylov, A. I. The spin–flip approach within time-dependent density functional theory: Theory and applications to diradicals. *The Journal of chemical physics* **2003**, *118*, 4807–4818.

(96) Casellas, J.; Bearpark, M. J.; Reguero, M. Excited-state decay in the photoisomerisation of azobenzene: a new balance between mechanisms. *ChemPhysChem* **2016**, *17*, 3068–3079.

(97) Lee, T. J.; Taylor, P. R. A diagnostic for determining the quality of single-reference electron correlation methods. *International Journal of Quantum Chemistry* **1989**, *36*, 199–207.

(98) Gozem, S.; Krylov, A. I.; Olivucci, M. Conical intersection and potential energy surface features of a model retinal chromophore: Comparison of EOM-CC and multireference methods. *Journal of chemical theory and computation* **2013**, *9*, 284–292.

(99) Asano, T.; Okada, T.; Shinkai, S.; Shigematsu, K.; Kusano, Y.; Manabe, O. Temperature and pressure dependences of thermal cis-to-trans isomerization of azobenzenes which evidence an inversion mechanism. *Journal of the American Chemical Society* **1981**, *103*, 5161–5165.
